# ES-ATRK: A Global Bundle Adjustment Initialisation Method for Event-Based Stereo Visual Inertial SLAM System Using Adaptive Threshold Robust Kernel Functions

**DOI:** 10.3390/s26103014

**Published:** 2026-05-10

**Authors:** Junyang Zhao, Han Yu, Zhili Zhang, Yaru Li, Huixin Zhu, Xingxu Yan, Jiayi Wang

**Affiliations:** School of Missile Engineering, Rocket Force University of Engineering, Xi’an 710025, China; zhaojy8611@outlook.com (J.Z.); zzl202@hhu.edu.cn (Z.Z.); 18215516817@163.com (Y.L.); hgdzhx@163.com (H.Z.); yanxx103@126.com (X.Y.); wjjy0111@163.com (J.W.)

**Keywords:** event-based stereo visual inertial SLAM systems, global bundle adjustment, robust kernels, adaptive threshold, ES-ATRK algorithm, reprojection residuals, absolute trajectory error

## Abstract

To address the issues of insufficient robustness, large depth recovery errors, and poor scene adaptability currently present in the initialisation phase of event-based stereo visual inertial SLAM systems, we propose a global BA initialisation method based on an adaptive threshold robust kernel function, ES-ATRK. The algorithm first achieves spatio-temporal fusion of events and visual features. Event features are triangulated to obtain depth values that serve as the 3D map, whilst visual features provide 2D observations; both modalities jointly feed the Structure from Motion (SfM) pipeline, laying the foundation for global bundle adjustment (BA) optimisation. The core contribution lies in incorporating a robust kernel function into the global BA to suppress outlier interference and in designing an adaptive thresholding algorithm that dynamically determines the kernel threshold. Furthermore, the algorithm calculates an initial threshold based on the quantile distribution of residuals prior to BA optimisation, combined with validity checks and a multi-round iterative smoothing adjustment strategy, thereby achieving scene-adaptive thresholding. In over 85% of the test scenes on the VECtor dataset, its localisation accuracy improved by at least 10% compared to existing mainstream event-based SLAM methods, such as ESVIO and USLAM. In high-dynamic scenes, its ATE performance is approximately twice that of mainstream models such as ESIO, and it maintains excellent positioning accuracy and stability of three-axis errors in generalisation tests on the HKU and MVSEC datasets. Furthermore, in the large-scale outdoor testing scenarios of the DSEC dataset, ES-ATRK also demonstrates superior feature tracking and trajectory estimation performance. This method effectively enhances the robustness of initialisation and depth recovery performance in event-based stereo visual inertial SLAM systems, reduces overall positioning error, and offers greater adaptability in challenging scenarios such as low-texture, high-dynamic, and HDR environments.

## 1. Introduction

In challenging motion scenarios, the combination of stereo event cameras, visual cameras, and inertial measurement units (IMUs) has been adopted by SLAM systems due to their complementary advantages at the data level. Although IMUs suffer from long-term drift, they can provide high-frequency, low-latency estimates of relative motion over short time intervals; visual sensors are prone to failure under low-texture or dynamic lighting conditions but can provide globally consistent observations, whilst event cameras generate asynchronous event streams with high dynamic range and low temporal latency by responding to logarithmic intensity changes. Event, visual, and inertial measurements are complementary and well-suited for sensor fusion. However, their combination suffers from significant depth recovery errors and poor scene adaptability during joint initialisation. In the application of event-based stereo visual inertial SLAM systems, failure to properly address these initialisation issues ultimately has a direct impact on the system’s positioning accuracy.

In event-based stereo visual inertial SLAM systems, the success of joint initialisation between event, vision, and the inertial measurement unit (IMU) depends largely on the effectiveness of event-vision data fusion. The essence of event-vision camera fusion lies in utilising observation data from both sources to reconstruct the scene via relevant algorithms, thereby employing this scene reconstruction information alongside IMU data for joint initialisation. Conventional SLAM systems typically employ monocular or stereo visual–inertial fusion for joint initialisation. Classic systems such as ORB-SLAM3 [1] and VINS-Mono [2] use visual SfM [3] to obtain an initial pose and feature points. They then fuse these with IMU pre-integration data to optimise scale, gravity vector, and IMU bias. However, these methods lack robustness in low-texture, high-motion, or occlusion scenarios. They are also susceptible to sensor noise during the initialisation phase. On the other hand, purely vision- or IMU-based approaches suffer from cumulative drift, requiring correction via loop closure detection or global optimisation.

Building upon the visual inertial SLAM systems described above, the event-based SLAM systems outlined below have begun to emerge. EVO [4] assumes that the scene is planar and parallel to the sensor; it then runs an attitude tracker to obtain an initial trajectory, and utilises this trajectory, event data, and the EMVS [5] method to compute an initial semi-dense 3D map, thereby completing system initialisation. On the other hand, EVIO [6] uses the collected event data as an initial time window, then selects corner points as features using FAST corner detection and the Shi-Tomasi method, and employs a two-stage EM algorithm to estimate optical flow and perform affine transformations. Finally, it combines extended Kalman filtering and IMU pre-integration to complete system initialisation. However, both methods suffer from insufficient robustness due to sensor noise, and their inability to immediately filter out certain anomalies results in inaccurate initial state estimates. Ultimate SLAM [7] collects IMU data to estimate the initial pose and the offsets of the gyroscopes and accelerometers, and generates motion-compensated event frames by synchronising standard frames with event spatio-temporal windows. Subsequently, based on FAST feature extraction and KLT tracking, the system effectively fuses event and image features with IMU pre-integration data to complete the initialisation. PL-EVIO [8] first uses visual SFM to obtain the camera pose, event corner points, and line feature positions, and then achieves self-initialisation of the unknown initial state through loose coupling with the IMU. ESVIO [9] first extracts visual and event corner features and performs spatio-temporal association matching. It then uses the RANSAC algorithm [10] to remove outliers and restores inverse depth via triangulation. Finally, it fuses IMU pre-integration data to construct spatial and temporal residual constraints within a sliding-window optimisation framework.

The SLAM systems described above share a common feature during initialisation: they utilise the SFM process for 3D structure recovery, a process also known as the Structure from Motion (SFM) algorithm. This algorithm comprises a series of steps, each of which involves corresponding sub-algorithms. Firstly, the system must perform feature matching using visual or event-based features; these features are not limited to corner points but also include point and line features, amongst others. Subsequently, triangulation is used to reconstruct a 3D point cloud, which is then employed to estimate the camera’s motion and pose. Next, errors are minimised through bundle adjustment [11], and finally, linear alignment is performed with pre-integrated IMU data to determine the scale and initial sensor offsets. The common advantage of the above systems is that they do not require scene prior knowledge and can autonomously reconstruct 3D structures and camera pose; however, their drawback is that they rely on texture and motion cues, which can easily fail in low-texture or high-speed scenarios. Furthermore, relying solely on visual information can lead to issues such as scale ambiguity and drift. Table 1 summarises the limitations of the more mature models currently used in visual inertial and event-based visual inertial SLAM systems, along with their corresponding initialisation algorithms.

As shown in Table 1, there has been limited research into optimising the SFM process during the initialisation of visual or event-based SLAM systems. Most existing methods rely on established algorithmic frameworks such as VINS-Mono; however, these established SLAM frameworks are often only suitable for non-challenging scenarios and fail to address issues such as insufficient robustness and inaccurate depth recovery in the system’s initialisation algorithms. Furthermore, these issues frequently compromise the system’s overall localisation accuracy; consequently, a method capable of providing high-precision initial pose estimates and minimal depth error during the initialisation phase is essential for SLAM systems. Throughout the entire SLAM process, if the system can be passed to the backend for global optimisation with a small depth error following initialisation, this will inevitably improve the system’s overall localisation accuracy.

Despite the complementary nature of event, visual, and inertial sensors, existing event-based stereo visual-inertial SLAM systems typically decouple the initialisation pipeline into two independent stages: spatio-temporal fusion of event and visual features using fixed geometric constraints, and standard bundle adjustment with a manually tuned, fixed-threshold robust kernel. This decoupled design suffers from a critical limitation: it ignores the heterogeneous and scene-dependent residual distributions of event features versus visual features. Event features exhibit higher noise levels and asynchronous temporal misalignment, whereas visual features demand fine geometric precision. A fixed Huber threshold cannot simultaneously accommodate these conflicting characteristics. Consequently, in challenging scenarios (e.g., low-texture, high-dynamic-range, or aggressive motion), a fixed low threshold prematurely discards valid event observations, whereas a fixed high threshold retains visual outliers, leading to progressive pose drift and amplified depth recovery errors. Furthermore, because SfM initialisation is an incremental process, residual magnitudes vary significantly across optimisation iterations; a fixed threshold is inherently incapable of adjusting to this temporal evolution. To bridge this gap, we propose ES-ATRK, a tightly coupled global BA initialisation framework that unifies event-visual spatio-temporal fusion with an adaptive threshold robust kernel. The central insight is to dynamically adjust the Huber threshold according to the quantile distribution of reprojection residuals prior to each BA round, thereby achieving scene-adaptive and iteration-adaptive robust optimisation.

**Figure 1:**
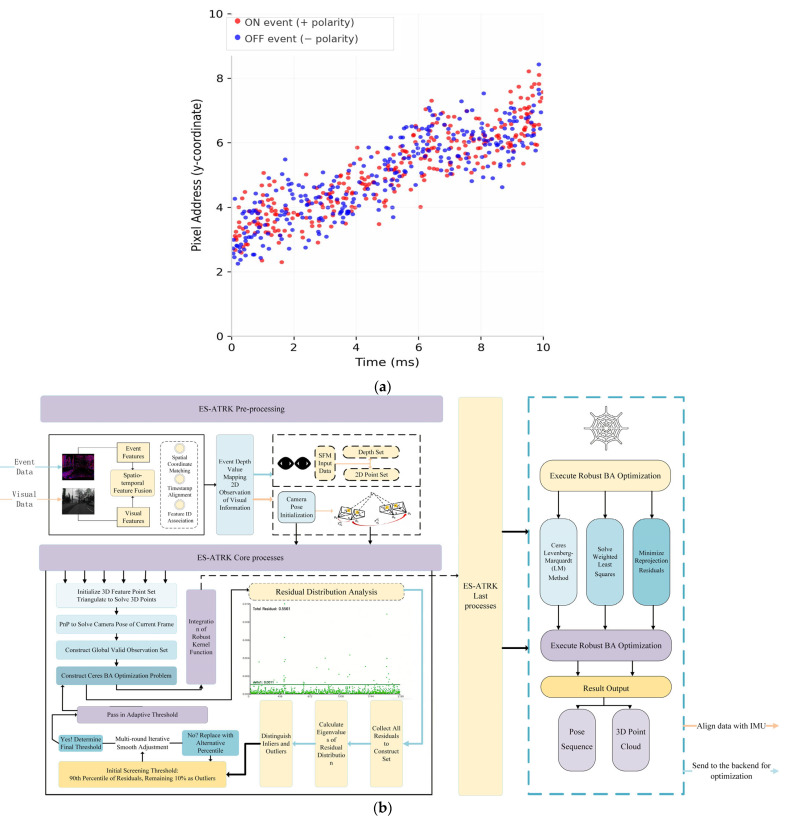
(**a**) Event Flow Diagram. (**b**) ES-ATRK Flowchart.

The research framework is shown in Figure 1b. Although robust kernel functions and bundle adjustment have been applied in existing visual and event-based SLAM systems, respectively, their application in event-based stereo-inertial initialisation remains limited to configurations with fixed thresholds and manual tuning, which decouple feature fusion from initialisation. For example, ESVIO and PL-EVIO employ standard bundle adjustment algorithms with fixed Huber or Cauchy kernels, while Ultimate SLAM relies on pre-integrated IMU constraints and fails to dynamically adjust the robust loss function based on the heterogeneous residual distributions of event and visual features. Nevertheless, their application in event-based stereo-inertial initialisation remains decoupled and static. Existing systems treat feature fusion and initialisation as independent stages, where event and visual features are fused according to a fixed workflow without any system feedback; furthermore, during the initialisation stage, the incorporation of robust kernels often takes the form of fixed thresholds, which cannot adapt to the heterogeneous noise characteristics of event and visual modalities. Therefore, the core design philosophy of ES-ATRK lies in how to effectively utilize the conflicting residual distributions generated by event and visual features simultaneously during initialisation. The novelty of ES-ATRK lies not in combining existing SLAM-related techniques, but in establishing a tightly coupled, self-adjusting optimization framework during the initialisation phase. Specifically, we introduce the following three innovations:

1. We design a feature spatiotemporal fusion mechanism. Unlike traditional methods that treat feature fusion as an independent preprocessing step, our method generates two complementary representations—event depth representations and visual 2D observations—and feeds both into the SfM pipeline. This ensures that both event and visual data are fully utilized in the subsequent BA optimization, thereby avoiding a single-modal optimization workflow.
2. We model SfM initialisation as a global BA problem with an adaptive threshold robust kernel. Its novelty lies not only in the introduction of the Huber kernel but, more importantly, in replacing the traditional fixed-threshold robust loss with a dynamic, scene-dependent threshold strategy. This strategy automatically determines the loss transition threshold based on the residual distribution before each optimization round, enabling the BA optimization to adapt to the current scene and filter out outliers.
3. We propose a convergent adaptive thresholding algorithm: after the system undergoes triangulation, we construct a set of residuals from valid reprojected points and filter in- and out-of-plane points based on the residual distribution. We then use the filtered threshold as the initial iteration value for boundary validity. Combined with a multi-round iterative smoothing mechanism, this ensures the algorithm evolves to a steady state, eliminating the influence of manual parameter tuning, Finally, this threshold is input into the Huber kernel of a global BA for optimization.

To the best of our knowledge, this is the first adaptive and robust BA mechanism applied to the initialisation stage of event-based stereo vision inertial SLAM.

## 2. Spatio-Temporal Fusion of Events and Visual Features

### 2.1. Spatio-Temporal Fusion of Event Features

Event cameras output asynchronous event streams rather than fixed-rate image frames. Consequently, traditional image-based instantaneous matching methods cannot be directly applied. Furthermore, matching based solely on temporal constraints is susceptible to temporal drift, sensor noise, and sensitivity variations. These factors can lead to mismatches. Therefore, extracting suitable event angle features and establishing spatiotemporal constraints is crucial for achieving data correlation between event angle features.

In our framework, stereo event streams generate two active event surfaces: a non-polar surface (SAE) and a polar time surface (TS) [14]. These surfaces store event pixel coordinates and timestamps. Through the TS, we convert asynchronous events into synchronous pseudo-images. This resolves the core issue that raw events cannot be processed directly by standard visual algorithms. We employ the Arc algorithm and event-based corner detection [15] on the SAE to extract event corners. Only events with timestamps close to the current time surface are retained. This preserves corner features within dense event streams. To mitigate noise, we further use a polarised time surface as a mask to filter valid corners.

(1) $$SAE\left(x,y\right)=\arg\max_{t\in T\left(x,y\right)}\left\{t|\left(x,y,t,p\right)\in E\right\}$$

SAE(x,y) represents the latest event timestamp recorded at pixel coordinates (x,y). Its core function is to bind the most recently triggered event time to each pixel, thereby achieving a temporal mapping from asynchronous events to spatial pixels. T(x,y) constitutes the collection of timestamps for all events triggered at the pixel coordinates (x,y). The quadruple (x,y,t,p) characterises the core attributes of an individual event. (x,y) denotes the pixel coordinates where the event occurred, p represents the event polarity (0/1, signifying brightness increase/decrease, respectively), and t denotes the timestamp for the individual event. Finally, E denotes the input set of raw stereo event streams.

Mapping SAE timestamps to grayscale values between 0 and 255, where lower values indicate newer events; simultaneously, TS integrates event polarity as a filtering mask to eliminate invalid or noisy events:

(2) $$TS\left(x,y,t_{cur}\right)=255\frac{t_{cur}-SAE\left(x,y\right)}{\Delta t_{\max}}$$

$TS(x,y,t_{cur})$ denotes the TS greyscale value of a pixel at the current timestamp; $t_{cur}$ represents the base timestamp at the current processing moment, serving as the reference time for the present temporal slice; $\Delta t_{\max}$ is a predefined hyperparameter measured in seconds, governing the TS greyscale range. Events occurring beyond this timeframe are mapped to 255 and deemed invalid.

The above process resolves the fundamental incompatibility between event and frame data. Event cameras report pixel-level brightness changes as they occur, but do not output complete images. Standard cameras capture full images at fixed intervals but provide no information about brightness changes between frames. The SAE records the most recent timestamp at each pixel, converting the temporal information of the event stream into a spatial map. The TS then assigns a greyscale value to each pixel based on how recently an event fired at that location. The resulting surface has the same spatial structure as a conventional grayscale image, which allows standard corner detection and optical flow algorithms to process event data directly. Brighter regions correspond to older events and darker regions correspond to newer events. The polarity mask removes events that would introduce noise into the surface, ensuring that only meaningful brightness changes contribute to subsequent feature extraction.

To establish a unified time reference that integrates time and space, we align the event stream with the timestamps of the video frames. We let $t_{frame}^{k}$ denote the timestamp of the kth visual frame. For each visual frame, we construct an event support window:

(3) $$W_{k}=\left\{e_{i}=(x_{i},y_{i},p_{i},t_{i})|t_{frame}^{k}-\Delta t\leq t_{i}\leq t_{frame}^{k}+\Delta t\right\}$$

Here, Δt=5 ms is the half-window duration; this value is chosen to match the temporal resolution of the visual exposure interval while ensuring sufficient event density for feature extraction. The event time surface is generated solely from events within Wk, effectively projecting the asynchronous event manifold onto the discrete timeline of visual frames. Because events and visual pixels are not captured simultaneously, each associated event-feature pair exhibits temporal misalignment:

(4) $$\delta_{t}=\left|t_{i}-t_{frame}^{k}\right|\leq \Delta t$$

Under the assumption of locally smooth camera motion, this temporal offset induces a spatial displacement proportional to the optical flow velocity:

(5) $$\delta_{x}=v_{x}\cdot \delta_{t}\begin{array}{cc} & \delta_{y}=v_{y}\cdot \delta_{t} \end{array}$$

To ensure that temporal misalignment does not corrupt the geometric consistency required for triangulation and BA, we enforce the synchronisation constraint:

(6) $$\max(\left|\delta_{x}\right|,\left|\delta_{y}\right|)<\varepsilon_{sync}$$

where $\varepsilon_{sync}$ = 0.5 pixels is the maximum allowable projection drift. Substituting a typical optical flow magnitude of $\left\|v\right\|\approx 50$ pixels/s, and a boundary of Δt=5 ms ensures that $\max(\left|\delta_{x}\right|,\left|\delta_{y}\right|)$ ≈ 0.25 pixels < $\varepsilon_{sync}$, satisfying this constraint. Event features that violate this boundary are discarded, ensuring that only temporally consistent observations participate in the subsequent SfM and BA processes.

Building on the above asynchronous event-visual synchronization and temporal error models, we employ the LK optical flow method to perform spatiotemporal feature fusion.

**Figure 2:**
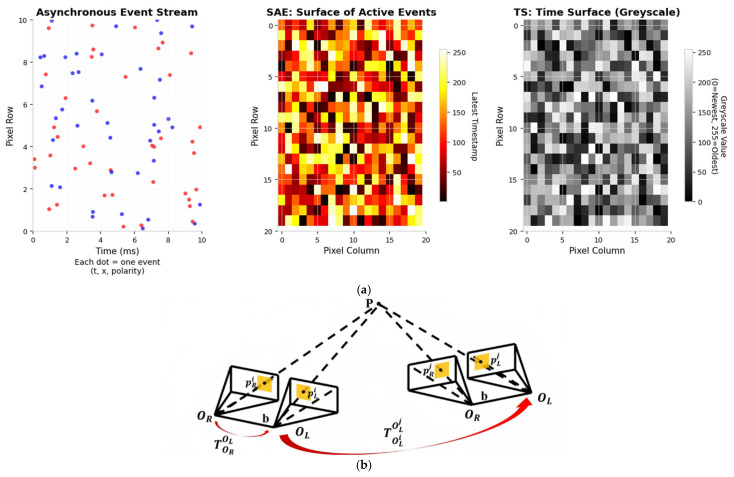
(**a**) Event-to-Image Conversion Pipeline. (**b**) Geometric principle of temporal and spatial event association. The colored dots represent event streams; red and blue indicate positive and negative polarity, respectively

Firstly, for newly arriving event streams, we utilise the LK optical method [16] to perform temporal tracking of existing event corner features, followed by spatial matching between the left and right event streams. Subsequently, we employ forward optical flow to predict feature positions and combine this with backward optical flow to verify consistency; this dual verification reduces the matching error rate. Finally, regarding temporal correlation, for the corner feature $F_{l}^{i}$ in the left event stream with timestamp i, we track its corresponding feature $F_{l}^{j}$ at the adjacent timestamp j using forward and reverse LK optical flow. Features that cannot be successfully tracked are immediately discarded to prevent the accumulation of errors. Regarding spatial correlation, for the left and right eye corner features $F_{l}^{i}$ and $F_{r}^{i}$ at the current timestamp i, spatial matching under epipolar line constraints is achieved on the temporal surface using forward and backward LK optical flow, ensuring that matched features lie along the epipolar lines. Figure 2b illustrates the geometric principle underlying event-based spatiotemporal correlation.

To ensure that the matching features between the left and right event streams lie along the epipolar lines, we perform spatial correlation whilst matching the time surfaces. The stereo event angle features $F_{l}^{i}$ and $F_{r}^{i}$ are instantaneously matched between the left and right temporal surfaces at the current time point i using forward and backward LK optical flow tracking, and we perform spatial correlation at every frame; simultaneously, for temporal correlation, we also use forward and backward optical flow to track the event angle features of the left event stream $F_{l}^{i}$ and $F_{l}^{j}$ at two consecutive timestamps i and j. The following formula represents our core assumption, namely that the TS pseudo-image after event transformation satisfies grey-level invariance, i.e., the TS grey-level value of the same feature remains unchanged at adjacent spatio-temporal positions:

(7) $$I_{e}\left(x,y,t\right)=I_{e}\left(x+dx,y+dy,t+dt\right)\Rightarrow I_{ex}u+I_{ey}v+I_{et}=0$$

$I_{e}\left(x,y,t\right)$ denotes the grey-scale value of the TS pseudo-image at position (x,y) and time t (corresponding to the TS grey-scale); $I_{ex}=\partial I_{e}/\partial x$ denotes the spatial gradient in the x direction, i.e., the horizontal gradient of the TS image; $I_{ey}=\partial I_{e}/\partial y$ denotes the spatial gradient in the y direction, i.e., the vertical gradient of the TS image; $I_{et}=\partial I_{e}/\partial t$ is the temporal gradient, i.e., the rate of change in grey-scale values between TS images at adjacent time slices; dx,dy is the displacement of features in pixel space, dt is the temporal displacement; u=dx/dt, v=dy/dt is the optical flow velocity, i.e., the pixel motion velocity in the direction of x,y.

Following the spatiotemporal association process described above, we use the RANSAC algorithm combined with fundamental matrix epipolar constraints to eliminate erroneous match pairs from the spatiotemporal association. We then perform two steps on the matched feature points: de-warping and pixel normalisation, before finally completing coordinate normalisation and triangulation to compute the inverse depth [17]. The formula for the epipolar constraint in the event-based matrix is

(8) $$p_{i}^{T}\overset{e}{F}p_{j}=0$$

where $p_{i}={\left(u_{i},v_{i},1\right)}^{T}$ denotes the homogeneous pixel coordinates of the matched feature points, $p_{j}$ and $p_{i}^{T}$ denote the homogeneous coordinates of the corresponding matched feature points, and $\overset{e}{F}$ is the event-based matrix, a 3 × 3 matrix describing the epipolar geometric constraints between the two views of the event camera [18].

The formulas for distortion correction and pixel normalisation are

(9) $${\tilde{p}}_{e}=undistort\left(p_{e}\right),X_{en}=\frac{\tilde{u}-c_{ex}}{f_{ex}},Y_{en}=\frac{\tilde{v}-c_{ey}}{f_{ey}},P_{en}={\left(X_{en},Y_{en},1\right)}^{T}$$

$p_{e}$ denotes the original pixel coordinates of the event corner; ${\tilde{p}}_{e}$ denotes the pixel coordinates after distortion correction (removing camera lens distortion); $f_{ex},f_{ey}$ denotes the camera intrinsic parameters, specifically the focal length; $c_{ex},c_{ey}$ denotes the coordinates of the camera’s principal point; $P_{en}$ denotes the normalised plane coordinates.

The geometric principles of triangulation-based depth recovery can also be seen in Figure 2a. At the timestamp i, the matching corner features of the 3D point p on the event camera’s imaging plane are $p_{L}^{i}$ and $p_{R}^{i}$, respectively. The inverse depth of p can be represented via epipolar geometry and the triangulation between $p_{L}^{i}$ and $p_{R}^{i}$, and $p_{R}^{j}$ and $p_{L}^{j}$ can also be used to invert the depth. Finally, we utilise the known two-frame stereo projection matrix $P_{0}=\left[R_{0}|t_{0}\right],P_{1}=\left[R_{1}|t_{1}\right]$ to match the normalised coordinates $u_{0}=\left(u_{0},u_{0}\right),u_{1}=\left(u_{1},u_{1}\right)$, thereby constructing the design matrix:

(10) $$\left\{\begin{array}{l} X_{n0}\cdot P_{0}\left(2,:\right)-P_{0}(0,:)\\ Y_{n0}\cdot P_{0}\left(2,:\right)-P_{0}(1,:)\\ X_{n1}\cdot P_{1}\left(2,:\right)-P_{1}(0,:)\\ Y_{n1}\cdot P_{1}\left(2,:\right)-P_{1}(1,:) \end{array}\right.\cdot P=0$$

We perform an SVD decomposition on the design matrix, take the last column of the V matrix as the homogeneous solution, and after normalisation, obtain the 3D point p, where $P_{0},P_{1}$ is the projection matrix, R is the rotation matrix, and t is the translation vector; together, these describe the projection relationship between the 3D point and the 2D pixel. $P_{0}\left(2,:\right),P_{0}\left(0,:\right),P_{0}\left(1,:\right)$ are the third, first, and second rows of the projection matrix $P_{0}$. $P=\frac{{\left(v_{0},v_{1},v_{2}\right)}^{T}}{v_{3}}$ i.e., the 3D point coordinates, used to calculate the inverse depth. $v_{0},v_{1},v_{2},v_{3}$ are the four elements of the last column of the V matrix following SVD decomposition.

After the inverse depth is recovered from the event data through the above process, it is fed into the SFM pipeline together with the 2D visual observations described in the next subsection.

The complete event processing pipeline performs three geometric operations in sequence. Firstly, temporal tracking establishes which corner in the current time surface corresponds to which corner in the previous time surface, determining the motion of each feature between moments. Secondly, stereo matching determines where the same corner appears in the left and right camera views at the same moment, which yields the disparity of the feature. Thirdly, triangulation converts this disparity into a depth value using the known baseline and calibration of the stereo rig. The epipolar constraint enforces that matching points must lie along a specific line in the opposite image, reducing the search from a two-dimensional region to a one-dimensional line and thereby decreasing both computational cost and mismatch rate. RANSAC further removes any matches that violate the epipolar geometry, ensuring that only spatially consistent features are used to compute depth. The final result is a set of three-dimensional points with recovered depth, each tied to a specific timestamp and camera pose.

### 2.2. Spatio-Temporal Fusion of Visual Features Based on Shi-Tomasi Corner Point Extraction

**Figure 3:**
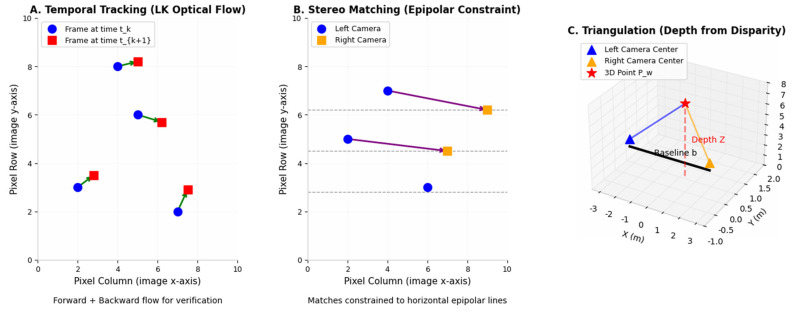
Spatio-Temporal Fusion Pipeline for Event and Visual Features. In the first figure, the green arrows indicate tracking from the current time step t to the next time step t + 1; in the second figure, the purple arrows indicate the registration between the left and right cameras.

Visual feature tracking follows the same geometric pipeline as event feature tracking, but begins with standard grayscale images rather than event time surfaces. The steps include LK optical flow tracking and fundamental-matrix epipolar constraints, as shown in Figure 3.

The core of visual feature processing is the extraction of stable corners from synchronised stereo grayscale images. These corners are tracked and matched, then converted into normalised 2D observations for SfM. In visual feature extraction, we employ the Shi-Tomasi corner point extraction method. According to Shi-Tomasi, a point is deemed a corner if the minimum eigenvalue of the evaluation matrix exceeds a threshold. The evaluation matrix is defined as

(11) $$M=\sum_{\left(x,y\right)\in W}\left[\begin{array}{cc} I_{x}^{2} & I_{x}I_{y}\\ I_{x}I_{y} & I_{y}^{2} \end{array}\right]$$

The criterion for determining a vertex is that $\min\left(\lambda_{1},\lambda_{2}\right)>\lambda_{th}$. Here, W is a local window centred on the pixel $\left(x,y\right)$; $I_{x}=\partial I/\partial x$ and $I_{y}=\partial I/\partial y$ are the gradients in the  x/y direction at the image location $\left(x,y\right)$; $\lambda_{1},\lambda_{2}$ are the two eigenvalues of the evaluation matrix M; and $\lambda_{th}$ is the corner threshold; pixels with values below this threshold are classified as non-corners, ensuring feature stability.

After extracting spatio-temporal gradient features, visual feature matching between frames and stereo matching is performed using Equation (8), based on the assumptions of grey-level invariance and local motion smoothing. In inter-frame tracking, we similarly utilise the LK pyramid optical flow method to perform feature tracking across consecutive frames for the left eye, combined with reverse optical flow for verification, thereby eliminating features with failed tracking. Finally, by checking whether features lie within the valid image region, we remove out-of-bounds points. In stereo matching, we reuse the LK pyramid optical flow method for feature tracking in both the left and right eyes. By tracking features from the left eye to the right eye and verifying them with reverse optical flow, we eliminate mismatched stereo vision features:

(12) $$I_{v}\left(x,y,t\right)=I_{v}\left(x+dx,y+dy,t+dt\right)\Rightarrow I_{vx}u+I_{vy}v+I_{vt}=0$$

where $I_{v}\left(x,y,t\right)$ denotes the grey-scale value of the visual image at position (x,y) and time t; $I_{vx}=\partial I_{v}/\partial x$ denotes the spatial gradient in the direction; $I_{vy}=\partial I_{v}/\partial y$ denotes the spatial gradient in the direction; $I_{vt}=\partial I_{v}/\partial t$ denotes the temporal gradient; dx,dy denotes the displacement of the feature in pixel space; dt denotes the temporal displacement; u=dx/dt and v=dy/dt denote the optical flow velocities.

Following stereo feature matching, we use the RANSAC algorithm in conjunction with fundamental matrix epipolar constraints to further eliminate outliers and remove invalid visual features, thereby ensuring a one-to-one correspondence between left and right eye features, and thus achieving spatiotemporal fusion of visual image features.

The formula for the visual fundamental matrix epipolar constraint is

(13) $$p_{i}^{T}\overset{v}{F}p_{j}=0$$

where $\overset{v}{F}$ is the visual image’s fundamental matrix, a 3 × 3 matrix describing the epipolar geometric constraints between the two visual views. After completing feature tracking and matching in stereo vision, operations such as distortion correction and pixel normalisation must be performed on the valid visual features, as per Equation (5) in the feature matching workflow, subsequently yielding coordinates $P_{n}$ in a unified format, i.e., 2D observations.

The formulas for distortion correction and pixel normalisation are

(14) $${\tilde{p}}_{v}=undistort\left(p_{v}\right),X_{vn}=\frac{\tilde{u}-c_{vx}}{f_{vx}},Y_{vn}=\frac{\tilde{v}-c_{vy}}{f_{vy}},P_{vn}={\left(X_{vn},Y_{vn},1\right)}^{T}$$

$p_{v}=\left(u,v\right)$ denotes the original pixel coordinates of the visual corner; ${\tilde{p}}_{v}=\left(u,v\right)$ denotes the pixel coordinates after distortion correction; $f_{vx},f_{vy}$ denotes the camera intrinsic parameters, i.e., the camera focal length; $c_{vx},c_{vy}$ denotes the principal point coordinates of the visual camera; $P_{vn}$ denotes the normalised visual plane coordinates.

In this process, unlike the event data, the visual 2D observation coordinates obtained after feature tracking, matching and normalisation using visual data are not triangulated; they are used purely for 2D observation.

The visual feature branch follows the same geometric pipeline as the event branch, but begins with standard grayscale images. Visual frames provide spatially dense and photometrically stable observations, because all pixels are captured simultaneously under the same exposure. However, this modality fails under motion blur or extreme illumination changes. The event branch compensates for these failures by providing features derived from brightness changes rather than absolute intensity values.

When both branches are combined, visual features supply accurate 2D reprojection constraints, whilst event features supply depth estimates through stereo triangulation. The two data streams are temporally aligned by anchoring the event accumulation window to each visual frame timestamp. This ensures that the depth map and the 2D observations describe the same scene state.

## 3. ES-ATRK Algorithm Workflow

Before proceeding to the SFM workflow within the ES-ATRK algorithm, the following two steps must be completed: solving for 3D points via triangulation and solving for camera pose using PNP. Only after these two foundational steps are completed will the obtained 3D points and camera pose undergo global BA optimization, the core of which lies in minimizing the reprojection error. In this algorithm, we employ the EPNP algorithm to rapidly solve for camera pose [19].

### 3.1. Robustness Design of the Algorithm

The core process of the entire ES-ATRK algorithm is the BA optimisation in SFM, and the core of BA optimisation is to minimise the reprojection error. Given the quaternion rotation vectors $q_{i}^{C}$ and $t_{i}^{C}$ of the camera in frame i, the 3D spatial coordinate point $X_{i}^{W}$, and the 2D observation $u={\left[u,v\right]}^{T}$, if the unit quaternion is $q={\left[w,x,y,z\right]}^{T}$ and the vector to be rotated is $p={\left[p_{x},p_{y},p_{z}\right]}^{T}$, then the rotated vector $p^{\prime }$ is

(15) $$p^{\prime }=q⊗\tilde{p}⊗q^{-1}$$

where ⊗ denotes quaternion multiplication, $\tilde{p}={\left[0,px,py,pz\right]}^{T}$ is the quaternion representation of the vector, and $q^{-1}={\left[w,-x,-y,-z\right]}^{T}$ is the inverse of the unit quaternion. The 3D coordinate point of the camera system is obtained via quaternion rotation and Equation (9):

(16) $$X_{i}^{C}=q_{i}^{C}\cdot X_{i}^{W}$$

Adding the translation vector $t_{i}^{C}$ yields the new 3D coordinate point in the camera system. $X_{i}^{C}$ can be expressed in the 3D coordinate system as $X_{i}^{C}={\left[X_{c},Y_{c},Z_{c}\right]}^{T}$, so the normalised reprojected point can be expressed as

(17) $$\hat{u}=\frac{X_{c}}{Z_{c}},\hat{v}=\frac{Y_{c}}{Z_{c}}$$

By calculating the 2D residual $r={\left[\hat{u}-u,\hat{v}-v\right]}^{T}$ and integrating the above steps, an exact representation of the residual can be obtained:

(18) $$r\left(q_{i}^{C},t_{t}^{C},X_{i}^{W}\right)=\left[\begin{array}{l} \frac{{\left(q_{i}^{C}\cdot X_{i}^{W}\right)}_{x}+t_{ix}}{{\left(q_{i}^{C}\cdot X_{i}^{W}\right)}_{z}+t_{iz}}-u\\ \frac{{\left(q_{i}^{C}\cdot X_{i}^{W}\right)}_{y}+t_{iy}}{{\left(q_{i}^{C}\cdot X_{i}^{W}\right)}_{z}+t_{iz}}-v \end{array}\right]$$

Here, ${\left(q_{i}^{C}\cdot X_{i}^{W}\right)}_{x}$ represents the x component of the vector after quaternion rotation, and $t_{ix}$ represents the x component of the translation vector. In our workflow, we utilise the Ceres solver to automatically compute the Jacobian matrix $J=\frac{\partial r}{\partial \theta }$ of the residuals with respect to each parameter block, thereby providing gradient information for the BA optimisation. BA optimisation is at the core of SFM; it minimises the global reprojection error by optimising all camera poses and 3D feature point coordinates. We incorporate the Huber robust kernel [20] to address outlier interference, thereby enhancing the overall robustness of the system during the initialisation phase.

The residual measures the geometric disagreement between a single 3D point and a single camera observation. If the estimated point position or camera pose deviates from the true value, the projected location shifts away from the measured pixel location. The objective function sums these deviations across all points and all cameras. Minimising this sum means finding the pose and point configuration that best explains all measurements simultaneously.

Without a robust kernel, every residual enters the sum as its squared magnitude. Squaring amplifies the influence of large residuals. Consequently, a single outlier can exert a disproportionately strong pull on the entire solution.

**Figure 4:**
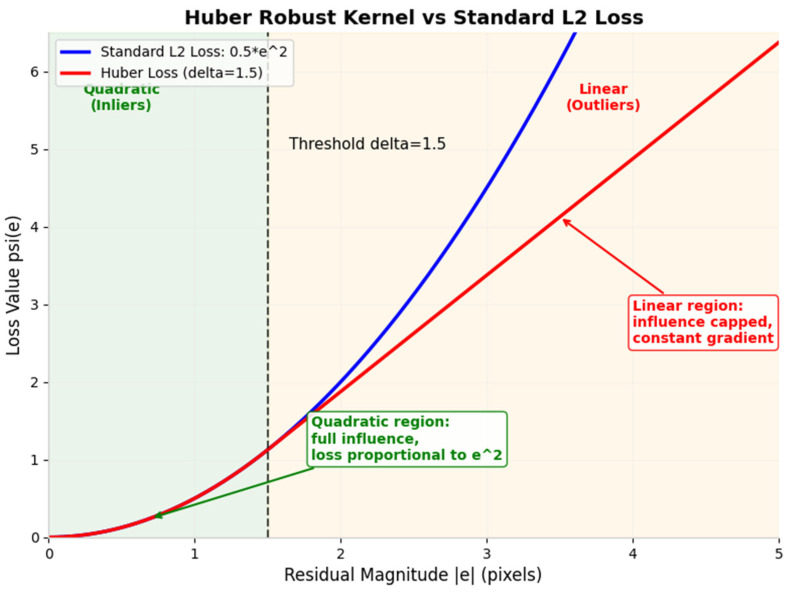
Huber Robust Kernel versus Standard L2 Loss.

Standard BA treats all camera poses and 3D points as optimisation variables, with the objective of minimising the sum of squares of the global reprojection error, as shown in Figure 4:

(19) $$\underset{{\left\{q_{i}^{C},t_{i}^{C}\right\}}_{i=1}^{n},{\left\{X_{j}^{W}\right\}}_{j=1}^{m}}{\min}{\sum_{i=1}^{n}\sum_{j=1}^{m}\delta_{ij}\cdot \left\|r_{i}^{j}\right\|}_{2}^{2}$$

where $\delta_{ij}$ is the observation validity indicator. To avoid scale ambiguity, in this optimisation problem, the rotation of frame l, the rotation of frame l, and the translation of the final frame are typically set as constants and used as the optimisation reference frame. A shortcoming of standard BA is that the sum-of-squares loss is highly sensitive to outliers, i.e., large residuals; a single outlier can skew the entire optimisation result. Our aim in incorporating a robust kernel function is to correct the residual loss, that is, to retain the sum-of-squares loss for small residuals at inner points whilst reducing the rate of increase in loss for outliers, thereby mitigating the impact of outliers on the optimisation. After incorporating the robust kernel function, the objective function for BA optimisation becomes the loss sum after applying the kernel function:

(20) $$\underset{{\left\{q_{i}^{C},t_{i}^{C}\right\}}_{i=1}^{n},{\left\{X_{j}^{W}\right\}}_{j=1}^{m}}{\min}\sum_{i=1}^{n}\sum_{j=1}^{m}\delta_{ij}\cdot \rho \left({\left\|r_{i}^{j}\right\|}_{2}^{2}\right)$$

where $\rho \left(s\right)$ is the robust kernel function. In this paper, the Huber kernel is used, and $s={\left\|r_{i}^{j}\right\|}_{2}^{2}$ is the L2 norm of the residuals. The Huber kernel is a piecewise function combining quadratic and linear components: it behaves as a quadratic function at interior points and as a linear function at outliers. We let the threshold of the Huber kernel be δ; its form is

(21) $$\mathrm{Huber}\left(r;\delta \right)=\left\{\begin{array}{cc} 0.5r^{2} & \left|r\right|\leq \delta \\ \delta \left(\left|r\right|-0.5\delta \right) & \left|r\right|\geq \delta \end{array}\right.$$

The loss function for the Huber kernel is

(22) $$\rho \left(s;\delta \right)=\left\{\begin{array}{cc} s & s\leq \delta^{2}\\ 2\delta \sqrt{s}-\delta^{2} & s\geq \delta^{2} \end{array}\right.$$

Within the domain of $s\leq \delta^{2}$, i.e., the interior region, this corresponds to the quadratic loss phase; within the domain of $s\geq \delta^{2}$, i.e., the exterior region, this corresponds to the linear loss phase. Furthermore, we must prove the continuity and differentiability of the kernel function itself, i.e., that the kernel function is continuous and globally differentiable at the threshold:

(23) $$\rho^{\prime }\left(s;\delta \right)=\frac{d\rho \left(s\right)}{ds}=\left\{\begin{array}{cc} 1 & s\leq \delta^{2}\\ \frac{\delta }{\sqrt{s}} & s\geq \delta^{2} \end{array}\right.$$

**Figure 5:**
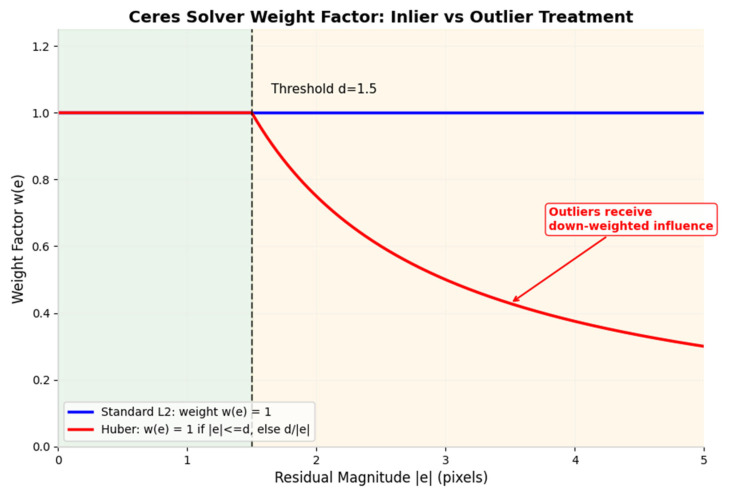
Ceres Solver Weight Factor for Inlier and Outlier Treatment.

Since for $\rho^{\prime }\left(s;\delta \right)$ at the threshold $s=\delta^{2}$ the left-hand limit equals the right-hand limit equals the function value and its derivative exists globally, the kernel function is continuous and globally differentiable at the threshold. Furthermore, as we utilised the Ceres solver in the global BA optimisation to automatically compute the Jacobian matrices of the residuals for each parameter block, we can, after incorporating the kernel function, analyse its weighting factors $w\left(s\right)$ within the Ceres solver to observe its influence on the optimisation directions for both interior and exterior points, as shown in Figure 5. The weighting factors $w\left(s\right)$ of the Ceres solver are defined as follows:

(24) $$w\left(s;\delta \right)=\frac{\rho^{\prime }\left(s\right)}{2s}=\left\{\begin{array}{cc} \frac{1}{2s} & s\leq \delta^{2}\\ \frac{\delta }{2s^{1.5}} & s>\delta^{2} \end{array}\right.$$

From the above equation, it can be observed that the weighting factor for outliers decreases monotonically as the residual increases; consequently, the contribution of outliers to the optimisation gradient is significantly reduced, preventing them from skewing the optimisation results. In contrast, the weighting factor for inliers remains unchanged, ensuring optimisation accuracy.

The Huber kernel treats small and large residuals differently. For residuals below the threshold, the kernel retains the squared loss. The derivative of the squared loss is proportional to the residual itself, so accurate measurements retain their full influence on the optimisation direction. For residuals above the threshold, the kernel switches to a linear loss. The derivative of the linear loss is constant, so the gradient contribution of an outlier does not increase with its magnitude.

The weighting factor in the Ceres solver implements this cap by scaling the residual before it enters the normal equations. An inlier retains its full weight of one. An outlier receives a reduced weight equal to the threshold divided by its residual magnitude. This scaling ensures that outliers participate in the optimisation only to the extent that they do not distort the final solution.

### 3.2. Adaptive Design of the Algorithm

In BA optimisation, if a robust kernel function is employed, the setting of its threshold is related to the reprojection error. Based on the assumption that the reprojection error follows a normal distribution, the threshold is typically set within a range of 1.5 to 3 times the standard deviation of the reprojection error. In challenging scenarios, a fixed threshold leads to three issues. Firstly, poor scene adaptability: a fixed threshold cannot respond to dynamic scene changes. In low-speed static scenes, it retains too many outliers, causing BA to favour erroneous observations. Secondly, incompatibility with heterogeneous features: event and visual features exhibit significantly different error characteristics, and a single fixed threshold cannot accommodate both. If a high threshold is set based on event features, minor errors in visual features will be overly tolerated, reducing localisation accuracy; if a low threshold is set based on visual features, reasonable errors in event features will be filtered out, resulting in an insufficient number of 3D points. Finally, a fixed threshold causes errors to accumulate and amplify. As SFM initialisation is an incremental process, a fixed threshold cannot be adjusted in tandem with the optimisation iterations. During the initial iterations, errors are relatively large, and a fixed low threshold will prematurely discard valid features; in the later stages, as iterative errors converge, a fixed high threshold fails to eliminate residual outliers, ultimately leading to global pose drift and a decline in localisation accuracy.

**Figure 6:**
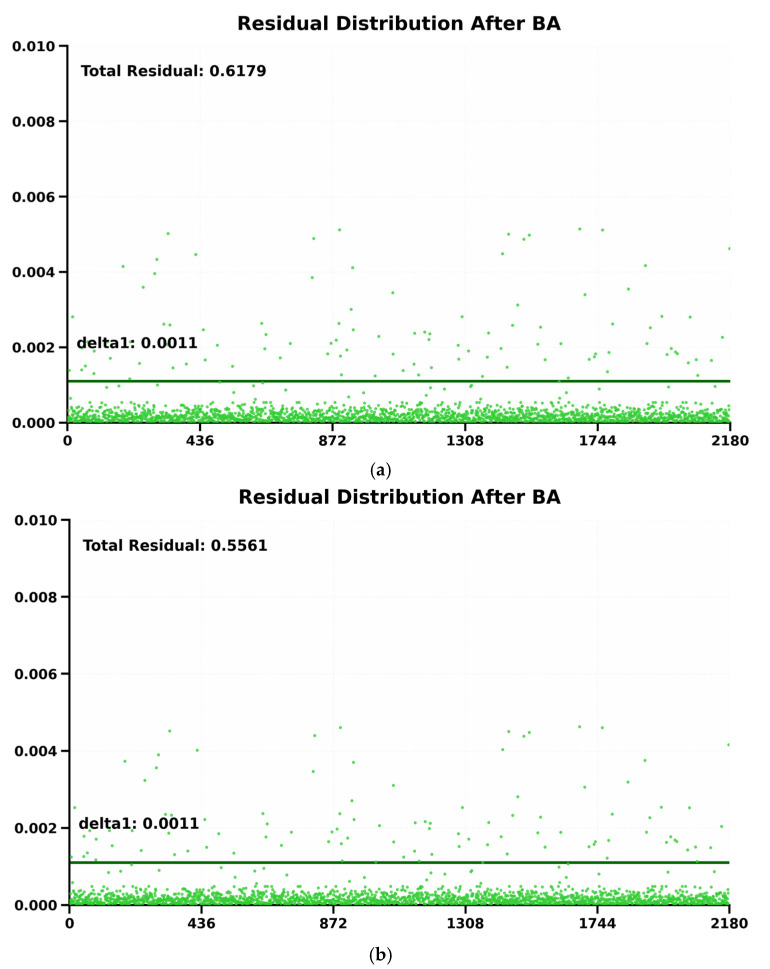
(**a**) Residual distribution plot optimized using the ES-ATRK algorithm. (**b**) Residual plot after optimization using standard BA.

The core idea of ES-ATRK is to flexibly adjust the Huber threshold according to the scene. In our algorithm, this threshold is determined by the residual distribution prior to each BA round. As shown by the threshold line in Figure 6a, a large number of residuals are concentrated at the bottom of the image; for this concentrated region, a quantile line delta1 is selected, and the value represented by delta1 serves as the threshold of the kernel function. Figure 6a,b show the residual distributions obtained using our ES-ATRK algorithm and a conventional BA optimisation algorithm based on a robust kernel function, respectively. As can be seen from the figures, the reprojection residuals following optimisation by the ES-ATRK algorithm (Figure 6a) are smaller than those of the conventional BA optimisation algorithm based on a robust kernel function, and the overall residual distribution is more concentrated, demonstrating the superior performance of our algorithm.

In the ES-ATRK algorithm we propose, the threshold of the robust kernel function is not a fixed value but is adaptively calculated from the residual distribution prior to BA optimisation. Furthermore, our algorithm undergoes multiple rounds of BA iteration to dynamically adjust the threshold, thereby avoiding manual parameter tuning, and finally performs a validity check to ensure the reasonableness of the threshold.

Firstly, the L2 norm of the globally valid residuals is pre-computed; that is, prior to BA optimisation, the L2 norm of the reprojected residuals for all valid observations ($\delta_{ij}$ = 1) is calculated, yielding the residual set:

(25) $$R=\left\{r_{1},r_{2},\ldots ,r_{N}\right\},{\begin{array}{cc} & r_{k}=\left\|r_{ik}^{jk}\right\| \end{array}}_{2}=\sqrt{{\left(\hat{u}-u\right)}^{2}+{\left(\hat{v}-v\right)}^{2}}$$

where N is the number of globally valid residuals and $r_{k}$ is the L2 norm of the th residual (in pixel units).

Next, the residuals are sorted and quantiles are calculated. The residual set R is sorted in ascending order to obtain an ordered sequence of residuals:

(26) $$R_{sort}=\left\{r_{\left(1\right)},r_{(2),}\ldots ,r_{\left(N\right)}\right\},\begin{array}{cc} & r_{\left(1\right)}\leq r_{\left(2\right)}\leq \ldots \leq r_{\left(N\right)} \end{array}$$

For any quantile ratio p∈(0,1], the precise formula for the quantile is

(27) $$\delta_{p}=r\left(\left\lfloor p\cdot N\right\rfloor \right)$$

Here, $\left\lfloor \cdot \right\rfloor$ performs a floor division; if p⋅N is an integer, the residual at the corresponding position is taken directly; if it is a decimal, the residual at the previous integer position is taken. We set the initial threshold to 0.9, i.e., $\delta =\delta_{0.9}=r\left(\left\lfloor 0.9N\right\rfloor \right)$, and use this as the initial iteration value, as this quantile ensures that 90% of the interior points fall within the quadratic region of the Huber kernel function, whilst only 10% of the exterior or outlier points fall within the linear region, thereby reducing their optimisation weight and balancing optimisation accuracy with robustness.

We emphasise that the 90th percentile serves exclusively as the initial iteration value of the threshold scheduler, not as a fixed operating point. Its statistical rationale rests on the standard inlier-rate assumption in robust geometric estimation. It is based on the standard inner-point assumption in robust geometric estimation.

(28) $$F_{inlier}(r)=CDF_{inlier}\begin{array}{cc} & F_{all}(r)=CDF_{all} \end{array}$$

We let $F_{inlier}(r)$ denote the cumulative distribution function (CDF) of inlier residuals, and $F_{all}(r)$ the CDF of all residuals. Under the typical RANSAC inlier-rate assumption ρ>0.85, the 90th percentile of the empirical residual distribution satisfies

(29) $$F_{all}^{-1}(0.90)\approx F_{inlier}^{-1}(0.90/\rho )$$

Since 0.90/ρ<1.06, meaning that this quantile lies within the high-probability region of the noise distribution, fewer than 0.1∗ρ<8.5% of the true inner points are misclassified into the linear region. Thus, the 90th quantile is established as the data-driven threshold, placing approximately 90% of the normal observations in the quadratic region and the remaining 10% of outliers in the linear region.

The choice of the 90th percentile, rather than the 85th or 95th, reflects a comprehensive consideration of the trade-off between bias and variance. A lower initial threshold would include more outliers in the quadratic region, increasing the risk of solution bias in high-dynamic-range scenes; a higher initial threshold, on the other hand, is overly conservative, as it increases variance by discarding noisy valid observations that are crucial in low-texture environments. However, since the threshold is updated iteratively through smoothing, the choice of initial percentile affects only transient behavior.

In Table 2, our ablation experiments confirm that thresholds of 85%, 90%, and 95% all converge to similar performance regions, indicating that the adaptive mechanism is robust to the initial percentile choice.

Next, a threshold validity check is performed. To prevent the threshold from failing due to an abnormal residual distribution (such as the majority of residuals being outliers), we incorporate a threshold validity check into the algorithm. The core validation formula is

(30) $$\delta_{\min}<\delta_{0.9}<\delta_{\max}\begin{array}{cc} & u_{r}=\frac{1}{N} \end{array}\sum_{k=1}^{N}r_{k},\begin{array}{cc} & \sigma_{r}^{2} \end{array}=\frac{1}{N}\sum_{k=1}^{N}\left(r_{k}-u_{r}\right)$$

$\delta_{\min}$ is the set minimum threshold, such as 0.1 pixels, which can be adjusted according to different scenarios. This value corresponds to the physical limit of reprojection error and is generally set to 5–10% of the residuals’ significant digits; residuals below this value are dominated by detector noise. The purpose is to prevent interior points from being incorrectly classified as exterior points rather than arbitrarily fixing the threshold boundary; $\delta_{\max}$ is the maximum threshold, such as 5 pixels, representing the maximum reprojection error caused by factors including camera calibration, lens distortion, and asynchronous timing errors. Residuals exceeding this threshold are considered gross errors, and this threshold is similarly set based on the distribution of reprojection residuals, generally at the 99th percentile or higher. Its purpose is to prevent outer points from being unduly weighted. The latter two are used to determine whether the residual distribution is normal. If the variance is too large, this indicates a high proportion of outliers, requiring adjustment of the kernel function threshold by substituting alternative percentiles such as $\delta_{0.85}$ or $\delta_{0.8}$. If the criteria are still not met, sequential search for a percentile threshold that satisfies the conditions should be continued. After determining the initial threshold, the threshold is dynamically adjusted through multiple rounds of BA iteration; that is, the residual distribution is recalculated after each round of adaptive BA optimisation, and the threshold is dynamically updated. The adjustment formula is

(31) $$\delta^{\left(k+1\right)}=\alpha \cdot \delta_{0.9}^{\left(k+1\right)}+\left(1-\alpha \right)\cdot \delta^{\left(k\right)}$$

$\delta^{\left(k\right)}$ is the threshold for the *k*th round of BA; $\delta_{0.9}^{\left(k+1\right)}$ is the 90th percentile calculated prior to the *k* + 1th round of optimisation (replaced with the corresponding value if using a different percentile); $\alpha \in \left(0,1\right]$ is the smoothing coefficient, typically set to α=0.8 to prevent optimisation oscillations caused by abrupt threshold changes; if the difference between $\delta_{0.9}^{\left(k+1\right)}$ and $\delta^{\left(k\right)}$ is less than the threshold ε, 5% of this difference is taken and adjustment is ceased, using the current threshold.

Setting the smoothing coefficient α=0.8 means that the threshold retains 80% of its previous value at each iteration, incorporating only 20% of the new quantile measurements. This setting is primarily intended to suppress transient fluctuations in the residual distribution, prevent the threshold from oscillating between consecutive iterations, and avoid misclassifying interior points as exterior points. These fluctuations are mainly caused by rounding errors in the median values during BA optimization or the absence of temporary features.

Experience shows that when α=0.8, monotonic convergence without oscillations can be achieved, converging to within 5% of the steady-state value in approximately three BA iterations, which aligns with the rapid reduction phase of the solver’s residuals. When α<0.6, it causes violent fluctuations in the threshold, thereby undermining the stability of BA optimization; when α>0.6, it slows down the convergence rate, leading to unnecessary computational costs before the threshold reaches the steady state for a specific scenario.

**Figure 7:**
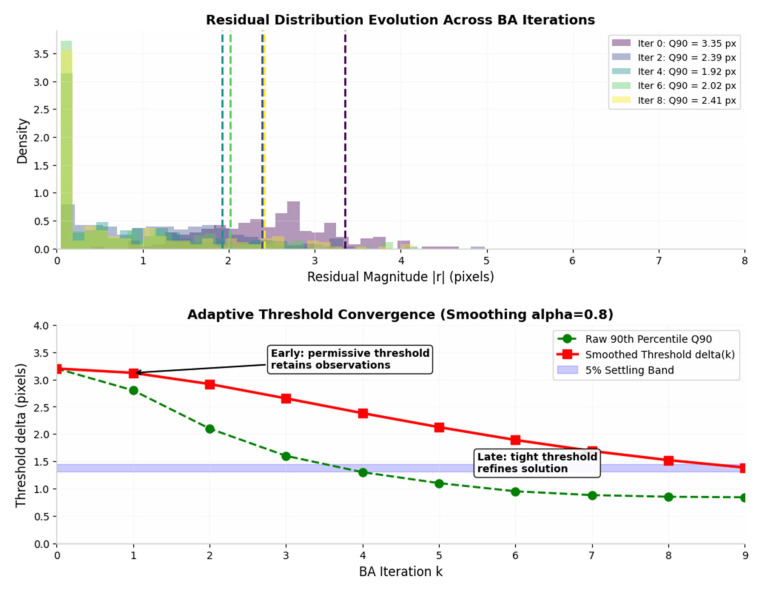
Adaptive Threshold Evolution Across BA Iterations.

**Figure 8:**
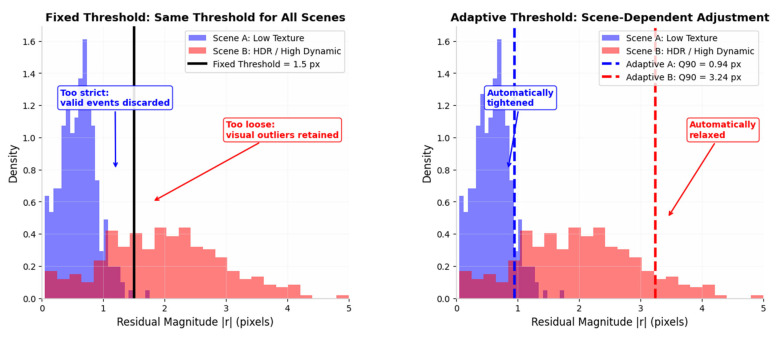
Fixed versus Adaptive Huber Threshold under Heterogeneous Scenes.

The smoothing update ensures that the threshold evolves gradually rather than jumping directly to the latest quantile value. If the threshold were allowed to change without restraint, a sudden shift in the residual distribution could reclassify a large number of inliers as outliers or vice versa. Such a reclassification would abruptly change the set of observations that contribute to the optimization, causing the cost landscape to fluctuate and preventing stable convergence. By blending the previous threshold with the newly computed quantile, the system maintains continuity in how observations are classified across consecutive iterations. The stopping criterion terminates the adjustment when the threshold enters a narrow neighborhood of its steady-state value. This indicates that the residual distribution has stabilized and that further changes to the threshold would produce negligible improvements in the objective function, as illustrated in Figure 7 and Figure 8.

Finally, we use the following formula to preliminarily evaluate the performance of the ES-ATRK algorithm:

(32) $$R=\sum_{k=1}^{N}r_{k}$$

Here, R represents the sum of global reprojection residuals, corresponding to the “Total Residual” value in the upper-left corner of Figure 6a,b. To intuitively illustrate the extent of the reduction in residuals, we use the residual reduction rate to characterize this effect:

(33) $$\eta =\frac{R_{init}-R_{final}}{R_{init}}\times 100\%$$

## 4. Experiment

To validate the effectiveness of the proposed algorithm, we conducted experiments on public datasets and evaluated the results using the EVO tool [21]. The datasets used for evaluation primarily include the following four: Vector [22], HKU [9], MVSEC [23], and DSEC [24], where the first three contain ground truth data, while the latter does not. For the first three datasets, we evaluated the algorithm’s performance through quantitative analysis. The EVO tool aligns the estimated trajectory with the ground truth using 6-DOF transformations and measures positioning accuracy via the Absolute Trajectory Error (ATE, %). In addition, we evaluated the algorithm’s performance using the root mean square error (RMSE, m) recovered after initialization, the residual reduction rate (η, %), and the initialization duration (t, ms).

The residual reduction rate η and the depth RMSE are two complementary metrics that characterise different stages of the initialisation pipeline. The residual reduction rate measures the relative decrease in the total sum of global reprojection residuals after BA. The residual reduction rate serves as a diagnostic indicator of BA optimisation health. A well-concentrated residual distribution—where most reprojection errors are driven into a low-magnitude regime with few persistent outliers—yields a more stable initial state than a scenario with a high reduction rate but a long tail of large residuals. It reflects the convergence quality of the robust kernel and the degree to which outliers are suppressed. The depth RMSE measures the Euclidean error between triangulated 3D points and ground-truth depths. It reflects the geometric fidelity of the recovered scene structure. A smaller depth RMSE directly determines the accuracy of the initial 3D map and camera poses. This provides more reliable reprojection constraints for subsequent frames and prevents trajectory error accumulation. The initialisation duration (t,ms) reflects computational efficiency and directly determines real-time performance.

Therefore, the optimal result is not simply maximizing the residual reduction rate but rather minimizing the root-mean-square depth error while further reducing residuals and ensuring a concentrated distribution and keeping the initialisation time within a reasonable range. The combined effect of these factors ensures that the back end receives a high-quality initialisation, thereby achieving higher positioning accuracy and real-time performance.

To ensure the fairness and rigor of the experiments, all evaluated methods were run under identical hardware and software conditions. The experimental hardware consisted of a laptop equipped with an Intel i7-13650HX CPU and 32 GB of RAM. Regarding software, the testing environment consisted of Ubuntu 20.04 and the Noetic version of ROS. For each comparison method, we used the official open-source implementation released by the original authors and adopted the default configuration parameters provided in their public code repositories. During testing, we did not perform any additional parameter tuning or scene-specific adaptation for any method, and all algorithms processed the same dataset files to eliminate configuration bias.

### 4.1. Ablation Experiment

To validate the appropriateness of the threshold parameters and kernel types selected in this paper, this section presents ablation experiments primarily based on the HKU dataset. These experiments not only compare the performance of the Huber, Cauchy, and Tukey kernel functions at a threshold of 1.0 but also evaluate the performance of the adaptive Huber kernel function proposed in this paper at three thresholds (95%, 90%, and 85%). Finally, control group data for the version without a kernel function are provided.

The HKU dataset was created by the University of Hong Kong to evaluate event-based visual inertial SLAM systems. The dataset was collected using various synchronized hardware sensors, including two DAVIS346 event cameras, each containing an event sensor, an image sensor, and an IMU. The dataset includes ground-truth annotations and covers a variety of fast-moving and highly dynamic scenes. Based on the experimental results, we compiled the data shown in Table 2.

Based on the data in the table, a comparison was conducted. Regarding ATE performance, regardless of the kernel function selected, the use of a fixed threshold causes the trajectory to diverge and the method to fail in the hku_agg_rotation test scenario, which is dominated by aggressive rotational motion. In contrast, our ES-ATRK method does not fail regardless of the initial threshold chosen and still achieves optimal ATE performance in the other two test scenarios. In terms of initialisation time, regardless of the threshold or kernel function selected by the system, its performance is comparable to that of the version without a kernel function, with both operating in the millisecond range, thereby meeting the real-time requirements of SLAM systems. Regarding depth error and residual reduction performance, since these factors interact and are reflected in the system’s ATE performance, our ES-ATRK method remains superior overall.

It should be noted that analyzing depth error or residual reduction performance in isolation has no practical significance, as examining only one of these metrics reflects merely numerical issues and does not indicate the algorithm’s inherent effectiveness. Furthermore, it should be noted that in the three test scenarios, the residual reduction in the Huber kernel function with a fixed threshold of 1.0 does not exhibit the data fluctuation issues seen with the other two kernel functions. However, this issue affects the system’s robustness and ultimately impacts ATE performance, which is why our ES-ATRK algorithm selects the Huber kernel function for adaptive threshold fusion.

**Figure 9:**
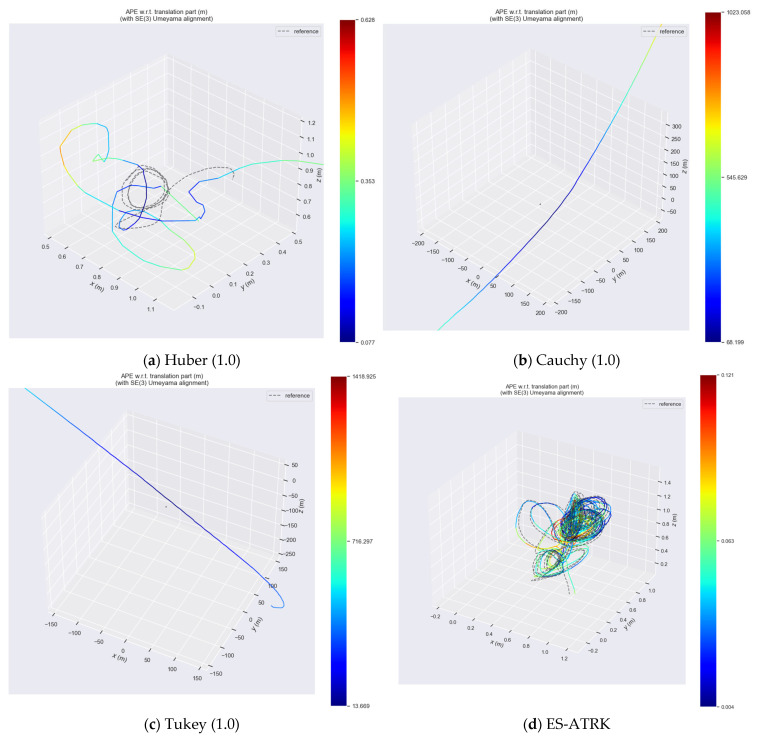
Trajectory heatmaps of various kernel functions and ES-ATRK in the hku_agg_rotation test scenario.

The ablation experiments described above rely solely on a single dataset and cannot effectively demonstrate the validity of our algorithm. To fully illustrate the effectiveness of our algorithm, we first conducted experiments on feature fusion, followed by full-scenario experiments, generalization experiments, and large-scale outdoor experiments using other datasets. Furthermore, we provided trajectory heatmaps for the hku_agg_rotation scenario using a kernel function with a fixed threshold and the ES-ATRK method. The results are shown in Figure 9, which clearly demonstrate that systems relying on a kernel function with a fixed threshold cannot adapt to challenging scenarios.

As can be seen in the figure, when the Cauchy or Tukey functions diverge due to the selection of a fixed threshold, their trajectories form curves that bear no relation to the ground truth. In contrast, when the Huber function uses a fixed threshold, its trajectory strives to converge toward the ground truth rather than diverging into a curve. This is because the residual filtering mechanism of the Huber kernel function is fundamentally different from that of the other two, preventing the trajectory from diverging into a curve. This is also why we chose the Huber kernel for our ES-ATRK algorithm.

### 4.2. Feature Fusion Experiment

To evaluate the performance of the feature fusion proposed by our algorithm, we conducted experiments on feature fusion effectiveness, using the total number of feature points, the number of 2D observations, and the number of depth observations as evaluation metrics. It should be noted that the algorithm’s effectiveness can only be demonstrated when the total number of feature points and the number of depth observations are sufficiently high, as only with a sufficient number of both can the algorithm successfully enter the SFM optimization phase. This experiment was conducted on the HKU datasets. The test results are shown in Table 3.

According to the data in the table, both the PL-EVIO and ESIO event-based visual-inertial SLAM systems outperform our algorithm in terms of the total number of feature observations prior to entering the SFM process. However, a higher total number of features is not necessarily better; as long as the number meets the initialisation requirements, any excess serves only to provide additional constraint information. In terms of the number of depth observations, our algorithm effectively utilizes the depth information from event data to obtain a reliable number of depth observations and more reliable depth constraint information.

The two systems we compared did not effectively utilize the depth information from event data prior to the initial SFM process; instead, they directly coupled it with visual data and fed it into the SFM to perform depth recovery operations such as PNP and triangulation. In contrast, our algorithm directly decomposes the two data types and treats the depth information recovered from the event data, together with the 2D observation information from the visual data, as a unified whole for subsequent optimization. This approach fully leverages information from both modalities, enhances feature fusion performance, and ultimately improves ATE performance.

To visually demonstrate the algorithm’s performance, we provide images showing feature tracking results on the hku_agg_rotation test dataset. Since PL-EVIO incorporates three modalities—event, visual, and inertial—visual images are displayed alongside the results when comparing it to our algorithm. In contrast, since ESIO only includes event and inertial modalities, only the event-based results are shown for comparison.

**Figure 10:**
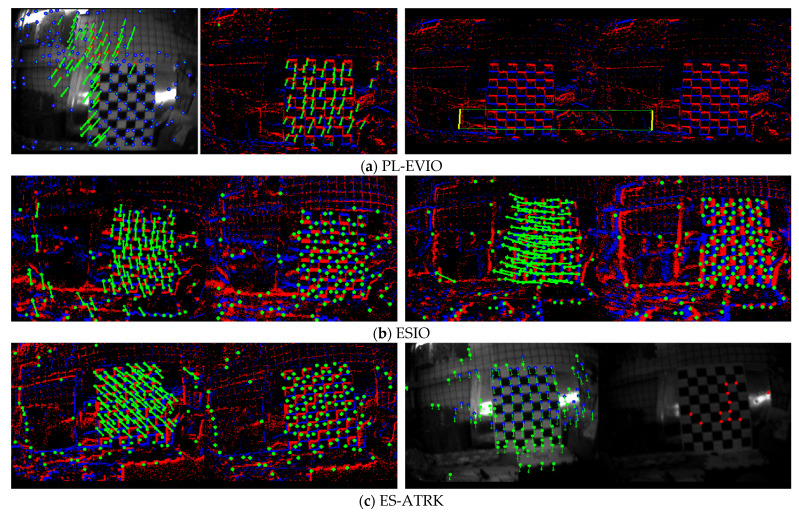
Feature Tracking Visualization. The green dots represent feature points, while the red and blue colors indicate the positive and negative polarities of the events.

As can be seen from the feature tracking results in Figure 10, PL-EVIO uses visual point features and event point-line features as its tracking model, but the density of its event feature points is far lower than that of the latter two. In contrast, ESIO’s event feature density is comparable to that of ES-ATRK, but it does not incorporate visual feature information. Finally, the ES-ATRK algorithm presented in this paper continues to provide critical data support for the SFM pipeline through effective feature tracking on the SAE, even when visual blurring occurs due to aggressive rotational motion, while simultaneously incorporating both event and visual data.

### 4.3. VECtor Full-Scenario Experiments

To comprehensively validate the robustness of the proposed algorithm, we selected the VECtor dataset for full-scenario testing. The VECtor dataset comprises data collected from a variety of hardware-synchronised sensors, including stereo event cameras, stereo reference cameras, RGB-D sensors, LiDAR, and IMUs. This dataset contains a wide range of motion scenes characterised by complex environments, varying lighting conditions, and different scales.

As shown in Table 4, our proposed ES-ATRK achieves the best results compared to the listed event-based algorithms. Although the ATE criterion of ESIO is slightly better than ours in certain sequences (e.g., corridors_dolly, school-dolly), our ES-ATRK yields more reliable and accurate results in most challenging scenarios involving HDR or aggressive motion. Furthermore, our proposed ES-ATRK is more accurate than the baseline model ESVIO, which may be attributed to the adaptive strategy of global BA optimisation in our algorithm, whereas traditional event-based methods (EVO, ESVO) failed in most sequences on the VECtor dataset. EVO and ESVO frequently fail on most sequences in VECtor, not because of insufficient parameter tuning or unfair experimental conditions. Rather, this reflects the inherent architectural limitations of single-modality event-based SLAM systems. EVO is a pure event-based monocular odometry method that assumes the scene has planar geometry and relies entirely on event-driven edge tracking, without fusing IMU or visual data. ESVO is a pure stereo event-based visual odometry system that processes asynchronous event streams but does not fuse standard camera frames or inertial measurement data. Both systems lack the ability to process complementary sensor modality data required to handle the challenging scenes found in the VECtor dataset, leading to system failures. These failures are inherent to single-modal event-based SLAM systems and cannot be mitigated by adjusting parameters alone.

Although ES-ATRK achieved the best ATE in most of VECtor’s test scenarios, three sequences are worth noting. In corridors_walk, ESIO reached 0.16%, while ES-ATRK reached 0.20%; in school-dolly, ESIO reached 0.25%, while ES-ATRK reached 0.30%; in corridors_dolly, the improvement over ESVIO was only 0.03 percentage points. These marginal effects or similar performance are not due to algorithm ineffectiveness, but rather to BA algorithm degradation issues triggered by specific scenarios. In corridors, highly repetitive parallel textures and a narrow field of view induce geometric degeneracy in triangulation. Consequently, the depth uncertainty along the corridor direction becomes poorly constrained. This reduces the effectiveness of reprojection-error minimisation regardless of the robust kernel employed. In school-dolly, the abrupt transition between indoor and outdoor areas causes transient sparsity in the event depth map. Meanwhile, in most normal scenes, the performance improvement is actually more significant than in fast scenes. In fast scenes, the residual distribution derived from event data is already reasonable, leaving limited room for improvement. In normal scenes, the visual-derived residual distribution is of lower quality than the event-derived one. The adaptive algorithm therefore has greater opportunity to improve performance by balancing the two modalities.

It is worth noting that even under these adverse conditions, ES-ATRK does not significantly lag behind the baseline models; its performance remains on the same order of magnitude, indicating that the algorithm’s adaptive mechanism provides a safety margin rather than causing a performance decline.

**Figure 11:**
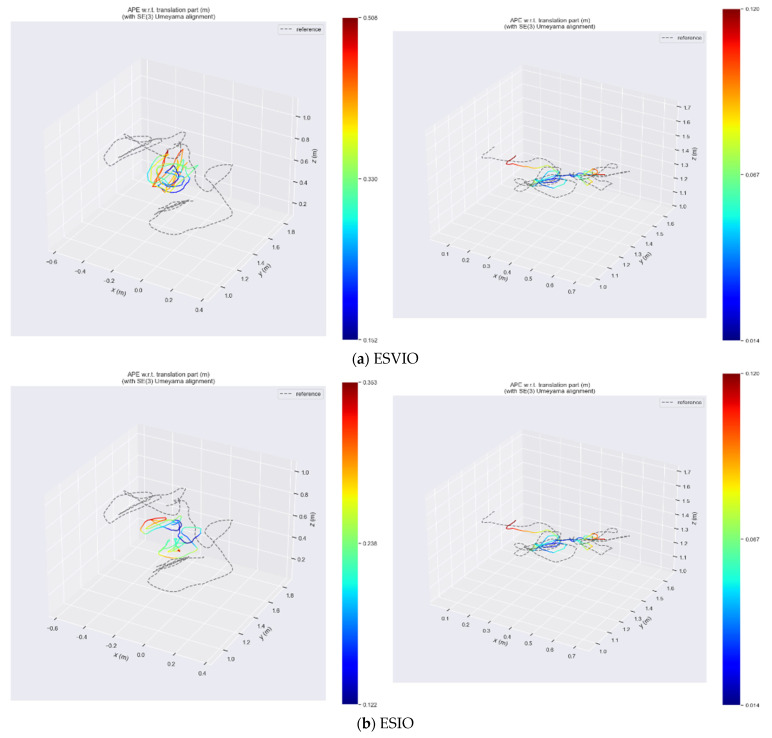

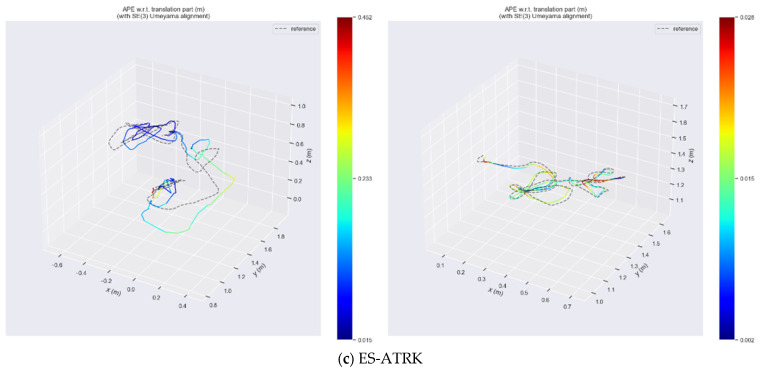
Heatmap of absolute trajectory errors for the ESVIO (**a**), ESIO (**b**), and ES-ATRK (**c**) algorithms in the mountain_normal and robot_normal test scenarios.

In the VECtor dataset, we selected two test scenarios—mountain_normal and robot_normal—to demonstrate the excellent performance of our algorithms; the former is a high-dynamic test scenario, whilst the latter represents an indoor enclosed environment. As can be seen from Figure 11, when compared with the ground truth trajectory represented by the dashed line, among the ESVIO, ESIO, and ES-ATRK algorithms, only the ES-ATRK algorithm exhibits the highest degree of fit to the ground truth trajectory whilst also having the smallest absolute trajectory error. In the mountain_normal test scenario, the ATE of the ES-ATRK algorithm was 1.29%, whilst that of ESVIO and ESIO was 4.17% and 2.79% respectively; in the robot_normal test scenario, the ATE of the ES-ATRK algorithm was 0.60%, whilst that of ESVIO and ESIO was 1.11% and 1.27%, respectively.

**Figure 12:**
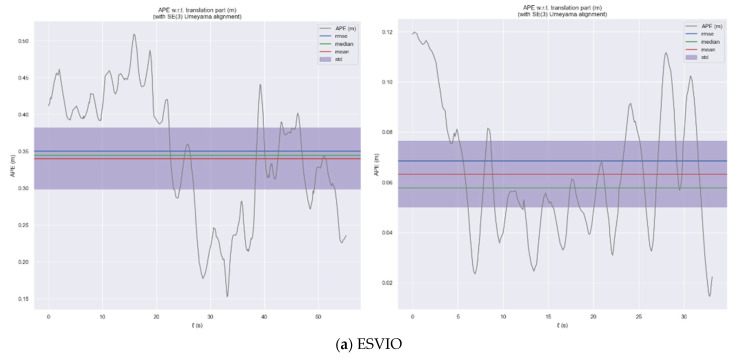

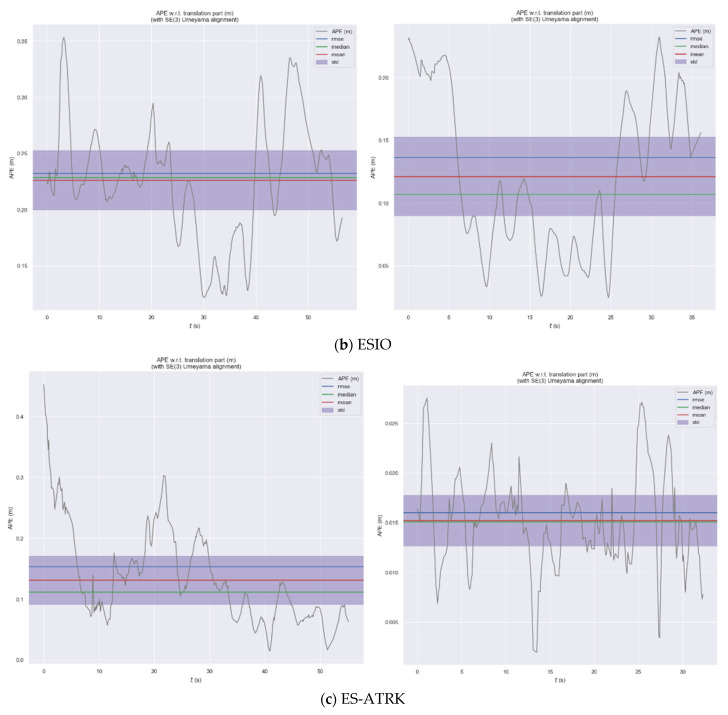
Time distribution curves of absolute trajectory error for the ESVIO (**a**), ESIO (**b**), and ES-ATRK (**c**) algorithms in the mountain_normal and robot_normal test scenarios.

In the following two test scenarios, the time distribution curves for the absolute trajectory error of the ESVIO, ESIO and ES-ATRK algorithms are shown in Figure 11 and Figure 12. It can be seen that in the high-dynamic test scenario “mountain_normal”, our ES-ATRK algorithm converges rapidly after a brief period of high error and remains stable overall, exhibiting a smaller and more stable ATE distribution compared to the other two groups. In the “robot_normal” test scenario, our ES-ATRK algorithm remains stable throughout the entire runtime, exhibiting not only smaller errors but also a more stable distribution.

**Figure 13:**
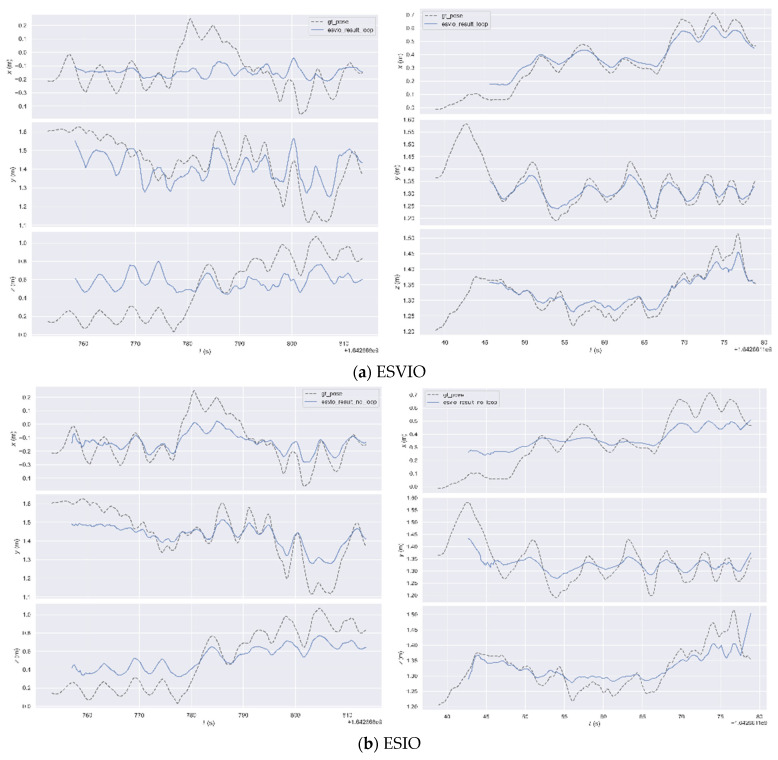

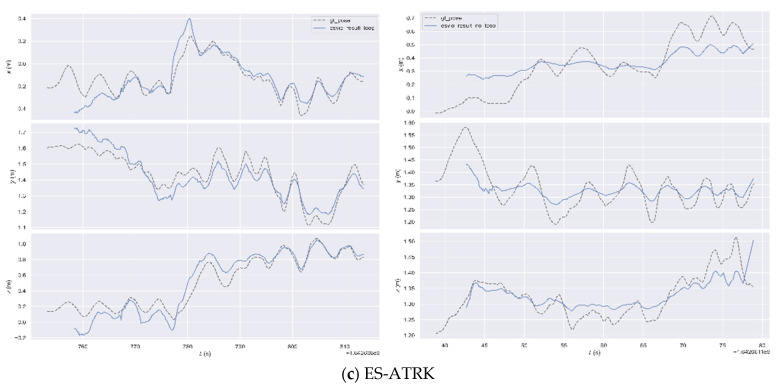
Three-axis distribution plots of absolute trajectory errors for the ESVIO (**a**), ESIO (**b**), and ES-ATRK (**c**) algorithms in the mountain_normal and robot_normal test scenarios.

In the following two test scenarios, we can also analyse absolute trajectory errors using three-axis error distribution plots. As shown in Figure 13, compared to ESVIO and ESIO, our ES-ATRK algorithm demonstrates superior stability and accuracy. Although we only presented error analysis plots for a subset of scenarios, the results in Table 4 indicate that, compared to most event-based SLAM algorithms, our proposed ES-ATRK algorithm achieves the best average absolute error (ATE) in the majority of test scenarios across the entire VECtor dataset, and the algorithm’s stability is also evident in the error distribution plots. Table 5 shows the combined mean squared error and residual reduction rates of the three algorithms after initialisation on the VECtor dataset. Our ES-ATRK algorithm also outperforms the other two algorithms in most test scenarios.

### 4.4. Generalisation Experiments

To validate the applicability of the proposed algorithm, we conducted experiments using the HKU and MVSEC datasets to assess its generalisation ability.

As shown in Table 6, similar to the results on the VECtor dataset, our proposed ES-ATRK algorithm still achieves better results than most event-based algorithms. Furthermore, since the selected scenes all involve challenging conditions such as high dynamic range, fast motion, or indoor flight, traditional event-based methods continue to perform poorly in most test scenarios. In contrast, our proposed ES-ATRK algorithm did not fail, which may be attributed to the robust and adaptive strategy employed in the global BA optimisation within the algorithm, as well as the full utilisation of event and visual data.

Notably, on the HKU dataset, the ATE value of the ES-ATRK algorithm is almost identical to that of ESVIO. This convergence is due to the dominant role of event features in the pose estimation process, which naturally leads to a compact residual distribution, enabling the adaptive thresholding method to achieve performance comparable to that of high-performing systems such as ESVIO and ESIO.

Therefore, these results do not undermine our argument but rather validate the design philosophy of the ES-ATRK adaptive mechanism. Specifically, when the scene produces low residuals, the initial threshold automatically converges to a fixed threshold range, thereby preventing the exclusion of valid observations due to unnecessary outliers. At the same time, ES-ATRK demonstrates robust performance in challenging scenarios such as Indoor_Flying1 and Indoor_Flying4.

**Figure 14:**
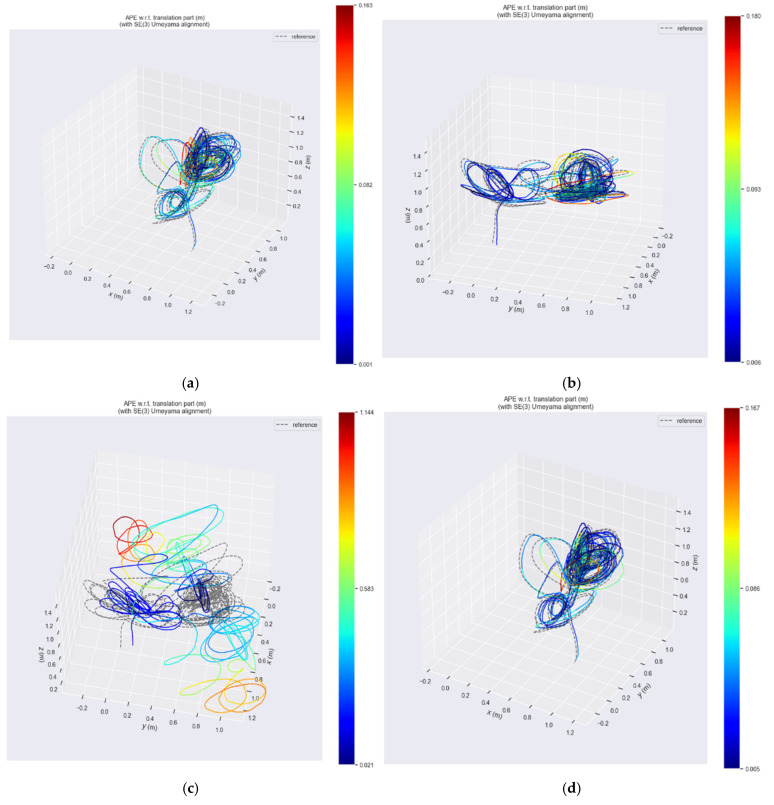
ATE heatmap of ESVIO (**a**), ESIO (**b**), PL-EVIO (**c**), and ES-ATRK (**d**) under hku_hdr_circle.

**Figure 15:**
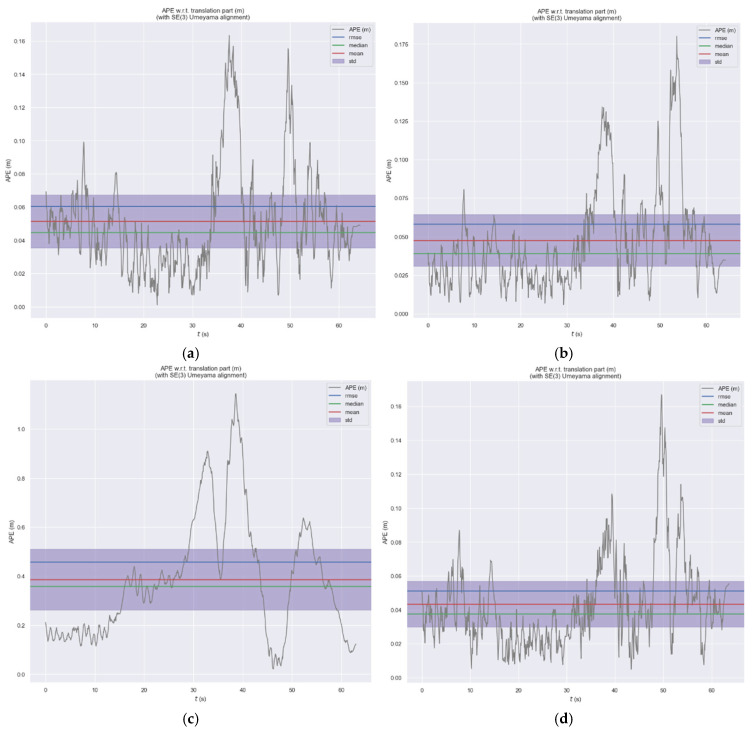
ATE plot of ESVIO (**a**), ESIO (**b**), PL-EVIO (**c**), and ES-ATRK (**d**) under hku_hdr_circle.

In Figure 14, we select the high-dynamic-range test scene hku_hdr_circle from the HKU dataset to demonstrate the performance of ES-ATRK. As can be seen from Figure 14 and Table 6, our ES-ATRK performs best among the four event-based multimodal algorithms, with an ATE of only 0.07%, which is 0.02 percentage points lower than that of the baseline model, ESVIO. Furthermore, in other test scenarios, the ATE performance of our ES-ATRK algorithm remains superior to the other methods mentioned in Table 4. Moreover, in Figure 14c, where PL-EVIO suffers from excessive trajectory errors due to the specific scenario, ES-ATRK still maintains an ATE of the same order of magnitude as the first two methods and is slightly superior to them. The time distribution curves of the absolute trajectory error for the above algorithms are shown in Figure 15. As can be seen from the figure, compared to ESVIO, ESIO, and PL-EVIO, our ES-ATRK algorithm exhibits a more concentrated and stable overall error distribution, with smaller values, indicating that our algorithm’s adaptive and robust strategies can enhance positioning accuracy and system stability.

Table 7 shows the root mean square error (RMSE) and residual reduction rate (η) of the three algorithms after initialisation on the HKU and MVSEC datasets. When considering both test datasets together, our ES-ATRK algorithm outperformed the other two algorithms in most test scenarios, thanks to our robust and adaptive global BA initialisation strategy.

### 4.5. Outdoor Simulation Experiments

Finally, we conducted outdoor simulation experiments on the DSEC dataset, which was collected using high-resolution stereo event cameras. In this dynamic scene, due to forward motion, few events are triggered at the centre of the field of view, presenting a challenge for SLAM algorithms that rely on event sensors. As the DSEC dataset does not provide ground truth, we could only perform a qualitative evaluation, presenting the estimated trajectories and image feature tracking performance as results.

**Figure 16:**
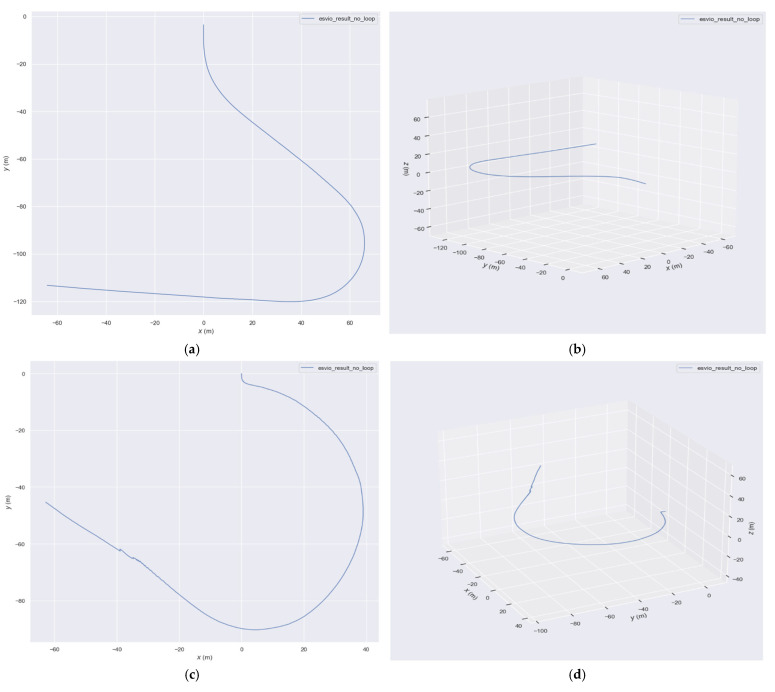

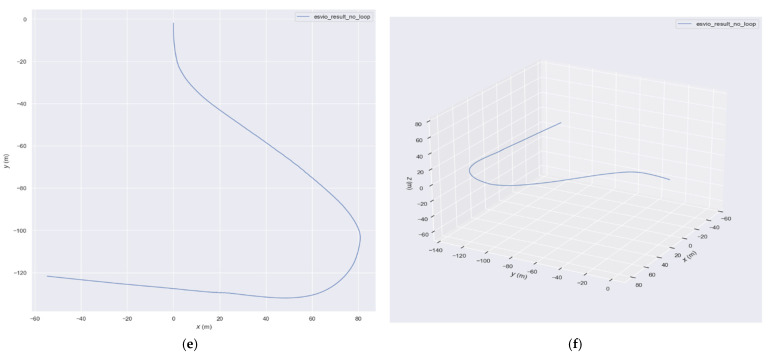
Ground relative trajectory estimated by ESVIO (**a**,**b**), ESIO (**c**,**d**), and ES-ATRK (**e**,**f**) in the Zurich_a outdoor scene.

We also present ground-truth trajectory plots for ESVIO, ESIO, and ES-ATRK. As the DSEC dataset does not provide ground-truth data, we can only provide relative estimated trajectories; however, our ES-ATRK still achieves satisfactory estimation results, as shown in Figure 16.

As can be seen from the figures, our ES-ATRK performs similarly to ESVIO in estimating ground trajectories, whilst ESIO lags behind both. This may be because ESIO only fuses event and IMU data, rather than integrating event, IMU and visual data as ES-ATRK and ESVIO do; consequently, ESIO exhibits a certain degree of deviation from the other two methods.

## 5. Conclusions and Future Work

To address the challenges in the initialisation of event-based stereo visual-inertial SLAM systems, we propose ES-ATRK, a robust real-time global BA initialisation method. The algorithm achieves spatio-temporal fusion between stereo event streams and visual frames. It enhances fusion performance by leveraging both visual 2D observations and event depth estimates. Subsequently, the fusion results are fed into the initial SFM process. Under the robust and adaptive strategy of global BA, the algorithm further suppresses reprojection and depth recovery errors while optimising the distribution of reprojection errors, thereby enhancing the system’s overall localisation accuracy and robustness. Furthermore, the algorithm achieves optimal ATE performance in most test scenarios, with performance improvements of at least 10% compared to the listed systems; in low-light scenes with a wide dynamic range, its ATE performance remains approximately twice that of models such as ESIO; in the VECtor full-scene and generalisation test scenarios, our ES-ATRK continues to demonstrate superior performance in terms of absolute trajectory and three-axis error distributions; and in large-scale tests without ground truth data, our algorithm still exhibits outstanding trajectory estimation performance.

**Figure 17:**
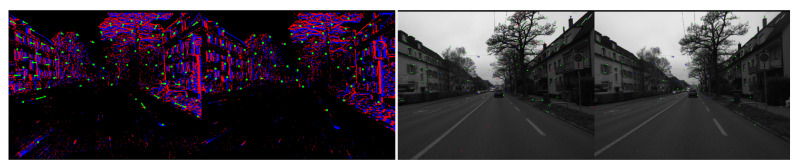
Feature tracking performance on the DSEC dataset. The green dots represent feature points, and the red and blue colors indicate the positive and negative polarities of the events.

Finally, although the proposed ES-ATRK algorithm achieves satisfactory results, it still has limitations. Specifically, as shown in Figure 17, in large-scale outdoor scenes, the feature extraction and matching processes may encounter issues such as a limited number of feature points and insufficient matching pairs. Furthermore, as shown in certain suboptimal test scenarios in Table 4 and Table 6, the adaptive threshold operates on the fused residual set without explicit per-modality weighting. Consequently, temporary degradation in one branch—whether the event branch or the visual branch—cannot be fully compensated for by the other branch. This results in optimisation outcomes that may be inferior to those achieved by standard BA optimisation. Future research could use this as a starting point for further exploration.

## Figures and Tables

**Table 1:** Key Research Findings Summary.

Category	Visual Inertial SLAM			Event-Based Visual SLAM		
	ORB-SLAM3 [1]	VINS-Mono [2]	SRVIO [12]	ESVIO [9]	PL-EVIO [8]	EVIO for UAV [13]
Algorithms	MAP Estimation + IMU Pre-integration	SfM + Linear Alignment + Gravity Optimisation	VINS-Mono Base + DNN Assistance	Stereo Event Matching + IMU Tight Coupling	Visual SfM + IMU Loose Coupling	Event Feature Tracking + IMU Pre-integration + Sliding Window
Whether SFM	Yes	Yes	Yes	Yes	Yes	NO
Limitations	Initialisation relies on static scene	Requires sufficient motion, pure rotation fails	GPU-dependent, dynamic scene sensitive	Motion compensation relies on backend output	SfM prone to failure in low-texture scenes	Event matching susceptible to high-speed motion

**Table 2:** Results of the Ablation Experiment.

Sequence		Nuclear Function Type			Threshold Type			Control Group
		Huber (1.0)	Cauchy (1.0)	Tukey (1.0)	Huber (95%)	Huber (90%)	Huber (85%)	
HKU(ATE)	hku_agg_rotation	fail	fail	fail	0.09	**0.09**	0.09	fail
	hku_hdr_circle	0.10	0.11	0.12	0.10	**0.09**	0.10	0.15
	hku_hdr_slow	**0.08**	0.11	0.11	0.11	0.10	0.10	0.10
HKU(t)	hku_agg_rotation	155	148	186	141	**110**	114	151
	hku_hdr_circle	192	**143**	155	184	184	190	150
	hku_agg_rotation	241	244	**237**	**238**	243	224	240
HKU (RESIDUAL RE)	hku_agg_rotation	43.53	43.23	**69.44**	46.80	43.52	41.99	43.53
	hku_hdr_circle	40.47	25.13	14.03	45.74	36.38	**49.43**	13.91
	hku_hdr_slow	50.14	52.38	53.38	**58.62**	55.15	55.62	52.38
HKU (DEPTH RMSE)	hku_agg_rotation	0.24	0.26	0.26	0.37	**0.26**	0.07	0.27
	hku_hdr_circle	1.08	0.49	0.52	0.55	**0.11**	0.47	0.52
	hku_agg_rotation	3.24	3.22	2.73	**1.34**	2.04	2.15	3.22

**Table 3:** Test results for Feature Fusion Experiment.

Sequence		PL-EVIO			ESIO			ES-ATRK		
		Total	valid_obs	valid_depth	Total	valid_obs	valid_depth	Total	valid_obs	valid_depth
HKU	hku_agg_rotation	355	355	0	269	215	0	158	155	23
	hku_hdr_circle	268	238	0	275	193	0	164	159	23
	hku_hdr_slow	232	221	0	293	203	0	178	168	18

**Table 4:** VECtor full-scene test results.

Sequence		EVO	ESVO	USLAM	PL-EVIO	ESIO	ESVIO	ES-ATRK
		ATE	ATE	ATE	ATE	ATE	ATE	ATE
VECtor	corner_slow	4.33	4.83	4.83	2.10	1.61	1.49	**1.36**
	robot_normal	3.25	failed	1.18	0.68	1.27	1.11	**0.60**
	robot_fast	failed	failed	1.65	0.17	0.24	0.10	**0.08**
	sofa_normal	failed	1.77	5.74	0.19	0.20	0.11	**0.10**
	sofa_fast	failed	failed	2.54	0.17	0.15	0.15	**0.13**
	mountain_normal	failed	failed	3.64	4.32	2.79	4.17	**1.29**
	mountain_fast	failed	failed	4.13	0.13	0.09	0.11	**0.08**
	hdr_normal	failed	failed	5.69	4.02	1.27	0.97	**0.84**
	hdr_fast	failed	failed	2.61	0.20	0.21	0.14	**0.13**
	desk-normal	failed	failed	2.24	3.66	1.78	0.46	**0.37**
	desk-fast	failed	failed	1.08	0.14	0.19	0.11	**0.11**
	corridors_walk	failed	failed	failed	0.92	**0.16**	0.21	0.20
	corridors_dolly	failed	failed	failed	1.58	0.57	0.26	**0.23**
	school_scooter	failed	10.87	6.40	1.30	0.92	1.05	**0.81**
	school-dolly	failed	2.91	failed	2.47	**0.25**	0.39	0.30

**Table 5:** Test results for initial depth error and residual reduction rate.

Sequence		ESIO	ESVIO	ES-ATRK	ESIO	ESVIO	ES-ATRK
		DEPTH RMSE			RESIDUAL RE (η)		
VECtor	corner_slow	0.82	0.61	**0.61**	28.66	40.00	**42.64**
	robot_normal	0.74	0.50	**0.40**	**53.90**	13.36	22.90
	robot_fast	0.17	0.11	**0.11**	57.94	66.84	**66.84**
	sofa_normal	0.38	0.36	**0.18**	12.23	**15.82**	14.87
	sofa_fast	0.63	0.54	**0.21**	41.19	42.68	**52.00**
	mountain_normal	0.63	0.61	**0.32**	42.92	43.66	**49.29**
	mountain_fast	0.70	0.62	**0.51**	42.72	42.66	**44.94**
	hdr_normal	**0.38**	0.63	0.42	**51.16**	28.37	47.89
	hdr_fast	0.38	0.36	**0.35**	33.66	33.12	**36.14**
	desk-normal	0.42	0.25	**0.08**	1.13	−58.40	**43.24**
	desk-fast	0.13	0.10	**0.10**	**43.58**	−12.19	2.46
	corridors_walk	**2.12**	25.05	4.18	27.73	34.86	**38.80**
	corridors_dolly	0.45	0.36	**0.35**	62.97	63.88	**67.29**
	school_scooter	**0.72**	0.78	0.75	42.14	31.43	**61.67**
	school-dolly	2.61	1.32	**1.29**	30.44	35.00	**44.50**

**Table 6:** ATE Results for Generalization Testing.

Sequence		EVO	ESVO	USLAM	PL-EVIO	ESIO	ESVIO	ES-ATRK
		ATE	ATE	ATE	ATE	ATE	ATE	ATE
HKU	hku_agg_rotation	failed	failed	failed	0.64	0.07	0.07	**0.07**
	hku_hdr_circle	failed	failed	0.92	0.82	0.19	0.09	**0.07**
	hku_hdr_slow	failed	failed	failed	0.12	0.08	0.08	**0.08**
MVSEC	Indoor_Flying1	5.09	4.00	failed	1.35	0.75	0.71	**0.55**
	Indoor_Flying2	failed	3.66	failed	1.00	0.74	0.76	**0.73**
	Indoor_Flying3	2.58	1.71	failed	0.64	0.40	0.44	**0.31**
	Indoor_Flying4	failed	failed	2.77	5.31	4.19	4.31	**3.17**

**Table 7:** Results for initial depth error and residual reduction rate in generalisation testing.

Sequence		ESIO	ESVIO	ES-ATRK	ESIO	ESVIO	ES-ATRK
		DEPTH RMSE			RESIDUAL RE (η)		
HKU	hku_agg_rotation	0.27	0.27	**0.25**	43.53	43.53	**43.52**
	hku_hdr_circle	1.62	1.08	**0.12**	25.12	**40.47**	36.38
	hku_hdr_slow	3.40	3.22	**2.04**	30.68	52.38	**55.15**
MVSEC	Indoor_Flying1	1.60	1.74	**1.14**	71.36	**71.37**	69.10
	Indoor_Flying2	2.67	2.68	**1.21**	**66.24**	58.12	55.13
	Indoor_Flying3	2.01	3.10	**1.04**	54.15	52.57	**62.28**
	Indoor_Flying4	1.37	0.62	**0.62**	61.77	64.76	**64.76**

## Data Availability

Data related to the current study are available from the corresponding author upon reasonable request. The codes used during the study are available from the corresponding author upon request.
